# A Genetic Progression Model of Braf^V600E^-Induced Intestinal Tumorigenesis Reveals Targets for Therapeutic Intervention

**DOI:** 10.1016/j.ccr.2013.05.014

**Published:** 2013-07-08

**Authors:** Roland Rad, Juan Cadiñanos, Lena Rad, Ignacio Varela, Alexander Strong, Lydia Kriegl, Fernando Constantino-Casas, Stefan Eser, Maren Hieber, Barbara Seidler, Stacey Price, Mario F. Fraga, Vincenzo Calvanese, Gary Hoffman, Hannes Ponstingl, Günter Schneider, Kosuke Yusa, Carolyn Grove, Roland M. Schmid, Wei Wang, George Vassiliou, Thomas Kirchner, Ultan McDermott, Pentao Liu, Dieter Saur, Allan Bradley

**Affiliations:** 1 Department of Medicine II, Klinikum Rechts der Isar, Technische Universität München, 81675, München, Germany; 2 German Cancer Research Center (DKFZ), German Cancer Consortium (DKTK), 69120 Heidelberg, Germany; 3 Wellcome Trust Sanger Institute, Genome Campus, Hinxton/Cambridge CB10 1SA, UK; 4 Instituto de Medicina Oncológica y Molecular de Asturias (IMOMA), 33193 Oviedo, Spain; 5 Instituto de Biomedicina y Biotecnología de Cantabria, 39011 Santander, Spain; 6 Department of Pathology, Ludwig-Maximilians-Universität, 80337 München, Germany; 7 Department of Veterinary Medicine, University of Cambridge, CB3 0ES Cambridge, UK; 8 Instituto Universitario de Oncología, Universidad de Oviedo, 33006 Oviedo, Spain; 9 Department of Immunology and Oncology, Centro Nacional de Biotecnología/CSIC, 28049 Madrid, Spain; 10 School of Pathology and Laboratory Medicine, University of Western Australia, WA 6009, Australia

## Abstract

We show that BRAF^V600E^ initiates an alternative pathway to colorectal cancer (CRC), which progresses through a hyperplasia/adenoma/carcinoma sequence. This pathway underlies significant subsets of CRCs with distinctive pathomorphologic/genetic/epidemiologic/clinical characteristics. Genetic and functional analyses in mice revealed a series of stage-specific molecular alterations driving different phases of tumor evolution and uncovered mechanisms underlying this stage specificity. We further demonstrate dose-dependent effects of oncogenic signaling, with physiologic Braf^V600E^ expression being sufficient for hyperplasia induction, but later stage intensified Mapk-signaling driving both tumor progression and activation of intrinsic tumor suppression. Such phenomena explain, for example, the inability of p53 to restrain tumor initiation as well as its importance in invasiveness control, and the late stage specificity of its somatic mutation. Finally, systematic drug screening revealed sensitivity of this CRC subtype to targeted therapeutics, including Mek or combinatorial PI3K/Braf inhibition.

## Highlights

• We present a progression model of BrafV600E-initiated serrated intestinal cancer
• Stage-specific genetic alterations drive sequential tumorigenesis
• Oncogenic signaling triggered by mutant Braf displays dose-dependent effects
• Large-scale pharmacologic profiling reveals new approaches for therapeutic targeting

## Graphical Abstract

**Figure:**
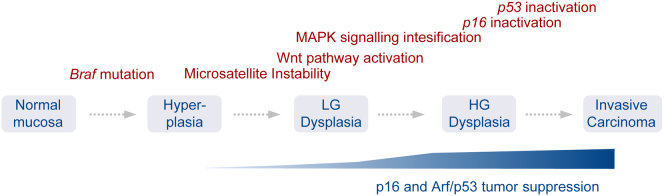

## Significance

**Dissecting the molecular pathways to colorectal cancer is essential to improve clinical classification and therapeutic stratification of this genetically heterogeneous disease. Here, we provide evidence for a *BRAF*-initiated pathway to intestinal tumorigenesis, which differs in molecular, morphologic, epidemiologic, and clinical aspects from the “classic” *APC-*induced pathway. Our studies describe key aspects of molecular tumor evolution, including tumor-driving alterations, the stage at which they occur, and mechanisms underlying their stage specificity. This knowledge together with systematic pharmacologic profiling revealed therapeutic opportunities for this tumor subentity. Colorectal cancer is the second most common malignancy in the Western world. The *BRAF* mutant subset alone has a higher incidence than many other human cancers. Our studies provide pathogenic insights and provide animal models for genetic/preclinical research.**

## Introduction

Colorectal cancer (CRC) initiation and progression is driven by a stepwise accumulation of genetic alterations (Fearon, 2011). There is however considerable genetic heterogeneity, and tumor subtypes evolve through different pathways. In the “classic” progression model, inactivation of the adenomatous polyposis coli *(APC)* gene is an early initiating event, followed by additional alterations, such as *KRAS* and *TP53* mutations (Fearon and Vogelstein, 1990; Fearon, 2011). These classic tumors are more often located in the distal colon and rectum and genetically they frequently have chromosomal instability (CIN). In this adenoma-carcinoma sequence, adenomatous polyps are the neoplastic precursor lesions of adenocarcinoma (Fearon, 2011).

Another type of polyp, the serrated polyp (formerly hyperplastic polyp) has long been considered to have no potential for neoplastic progression. This concept was challenged by the observation of cancers developing in patients with hyperplastic polyposis syndrome (Torlakovic and Snover, 1996) or in sporadically occurring hyperplastic polyps (Torlakovic et al., 2003). These studies recognized that lesions classified until then as hyperplastic polyps represent in fact several subentities, some of which are precancerous. Since then, numerous reports have confirmed and extended these findings and conclusions (for recent reviews see Noffsinger, 2009; Rex et al., 2012; Bettington et al., 2013), and consequently hyperplastic polyps have been renamed as serrated polyps. The latest World Health Organization classification distinguishes three categories of serrated polyps: hyperplastic polyps (HPs), sessile serrated adenomas (SSAs), and traditional serrated adenomas (TSAs; Snover et al., 2010). The major histologic feature of all serrated polyps is the saw-toothed (serrated) infolding of the crypt epithelium.

HPs are characterized by an expanded proliferation zone, but do not have architectural changes or dysplasia. They account for at least 80%–90% of serrated polyps and can be found in 20% of adults in Western populations. Ninety percent of HPs are small (<0.5 cm) lesions in the rectosigmoid and have little potential for malignant progression. However, large left-sided HPs (>0.5 cm) and right-sided HPs of any size have been associated with increased cancer risk and their removal is now being recommended (Rex et al., 2012).

SSAs resemble HPs, but can be distinguished pathologically by their abnormal architectural features, including dilated and branched crypts. Increased proliferation can be observed, but typically there is no or only minimal dysplasia. TSAs are characterized by a tubulovillous architecture and eosinophilic epithelium with serration and uniform cytologic atypia (dysplasia). Both SSAs and TSAs have a significant risk for malignant transformation and their removal is therefore recommended (Rex et al., 2012).

It has been estimated that up to 30% of colorectal cancers evolve from these precursor lesions through a “serrated pathway” (Rex et al., 2012). Serrated cancers are considered to differ not only morphologically, but also in their genetic characteristics from “classic” tumors arising through the adenoma-carcinoma sequence (Noffsinger, 2009; Rex et al., 2012; Bettington et al., 2013). Serrated polyps predominantly have mutations in either *BRAF* or *KRAS* but less frequently in *APC*. They typically lack CIN but often exhibit high level microsatellite instability (MSI-H) and extensive DNA methylation of CpG islands (CIMP-H). Based on these genetic alterations, Jass proposed that most serrated tumors can be classified into three major subtypes: (1) *KRAS* mutant, CIMP-Low, MSS/MSI-Low; (2) *BRAF* mutant, CIMP-H, MSI-H; and (3) *BRAF* mutant, CIMP-Low, MSS/MSI-Low (Jass, 2007).

After the first reports of *BRAF* mutations in colorectal malignancy (Davies et al., 2002; Rajagopalan et al., 2002), it has soon been recognized that *BRAF* alterations are strongly associated with right-sided sessile cancers and its serrated precursor lesions HPs and serrated adenomas (Chan et al., 2003; Yang et al., 2004; Kambara et al., 2004; Spring et al., 2006). The most frequent somatic alteration in *BRAF* is a point mutation (T1799A encoding BRAF^V600E^), which results in a several hundred-fold increased activity of the protein’s kinase domain. This causes sustained activation of the MEK1/2 → ERK1/2 mitogen-activated kinase (MAPK) signaling cascade (Davies et al., 2002), a pathway that controls a wide range of physiologic and tumor-promoting processes, including self-renewal, proliferation, senescence, apoptosis, invasion, and metastasis. To study the role of BRAF^V600E^ in intestinal tumorigenesis, we developed conditional *Braf*^*V637E*^ knockin mice, in which mutant *Braf* can be expressed in a tissue-specific manner from its endogenous locus.

## Results

### BRAF^V600E^ Initiates a Serrated Pathway to Intestinal Tumorigenesis

**Figure 1:**
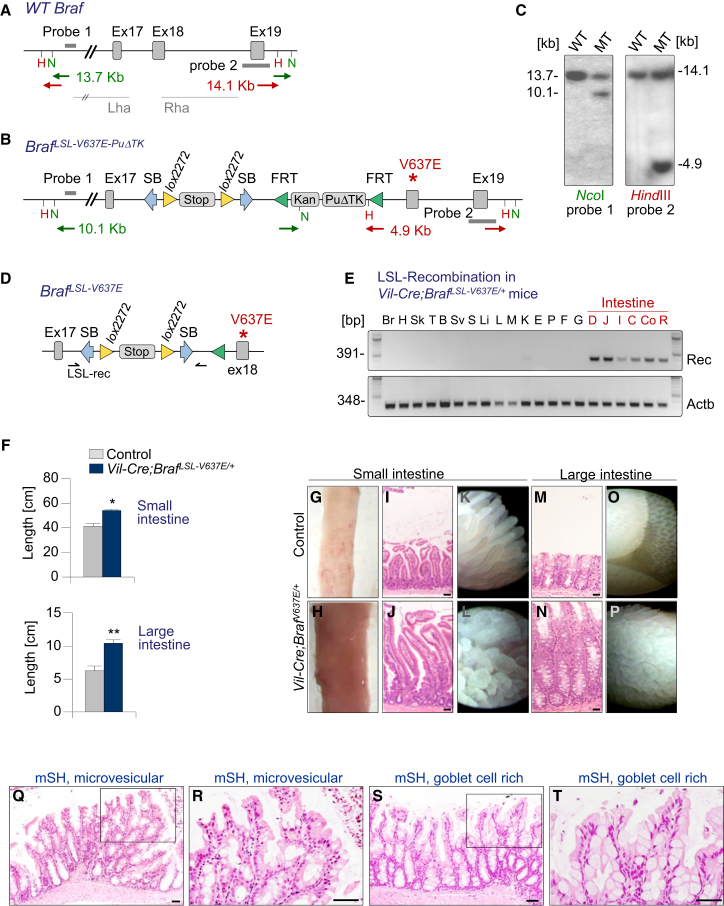
A Mouse Model of Braf^V600E^-Induced Intestinal Pathology (A–D) Knockin strategy of the *Braf*^*V637E*^ allele. Wild-type mouse *Braf* locus. Lha/Rha, left and right homology arms (A). Targeted *Braf*-locus (B). The Lox-STOP-Lox cassette has an Engrailed-2 splice acceptor and 4 SV40 polyadenylation sites. It is flanked by *Sleeping Beauty* inverted terminal repeats permitting *SB* transposase-dependent V637E activation, a feature not exploited in this study. Southern blot confirming correct targeting (C). F1 males were mated to *Rosa26-FlpE* females to remove the FRT flanked puromycin resistance cassette (PuΔTK), producing offspring with the *BRAF*^*LSL-V637E*^ conditional allele (D). (E) Villin-Cre-induced recombination of the STOP cassette in *Braf*^*LSL-V637E/+*^ mice. Br, brain; H, heart; Sk, skin; T, testis; B, bladder; SV, seminal vesicle; S, spleen; Li, liver; L, lung; M, muscle; K, kidney; E, esophagus; P, pancreas; F, forestomach; G, glandular stomach; D, duodenum; J, jejunum; I, ileum; C, coecum; Co, colon; R, rectum. (F–P) Pronounced generalized intestinal hyperplasia in *Vil-Cre;Braf*^*LSL-V637E*/+^ mice. Length of the small (SI) and large intestine (LI) in *Vil-Cre;Braf*^*LSL-V637E*/+^ mice and *Braf*^*LSL-V637E/+*^ control animals (F). Error bars, SEM; n > 15 per group; ^∗^p < 0.05; ^∗∗^p < 0.001 by t test. Thickening and elongation of intestines in *Vil-Cre;Braf*^*LSL-V637E*/+^ mice (G–P). Representative macroscopic (G/H), microscopic (I/J/M/N), and endoscopic (K/L/O/P) images of SI and LI from *Braf*^*LSL-V637E/+*^ controls and *Vil-Cre;Braf*^*LSL-V637E*/+^ mice. Scale bars, 50 μm. (Q–T) Serrated hyperplasia in *Vil-Cre;Braf*^*LSL-V637E*/+^mice. Microvesicular hyperplasia in the SI showing crypt elongation and serrated epithelium. mSH, murine serrated hyperplasia (Q and R). Goblet cell-rich hyperplasia in the large intestine with crypt elongation, sparsely serrated epithelium and large numbers of goblet cells in the large intestine (S and T). Scale bars, 50 μm. See also Figure S1.

To examine the effect of *Braf*^*V600E*^ in the intestine, we created a *Braf* knockin allele, which can be activated by Cre, leading to the production of the V637E mutant Braf protein. *Braf*^*V637E*^ in mouse exon 18 is at the orthologous position of the human *BRAF*^*V600E*^ mutation affecting exon 15. In the absence of Cre, a Lox-Stop-Lox cassette located in intron 17 prevents expression of the mutant allele (Figures 1A–1D). To direct mutant Braf expression to the intestine, we used *Villin-Cre* (*Vil-Cre*) mice in which Cre is broadly expressed in epithelia of the small and large intestine (Madison et al., 2002). In *Vil-Cre;Braf*^*LSL-V637E/+*^ mice the stop cassette at the *Braf* locus is excised specifically in the intestine but not in other organs (Figure 1E). The murine *Braf* ^*LSL-V637E*^ allele is a knockin allele and is thus expressed from the endogenous *Braf* locus at physiologic levels.

All *Vil-Cre;Braf* ^*LSL-V637E*/+^ animals developed lifelong persistent generalized crypt hyperplasia affecting nearly every crypt, leading to significantly elongated and thickened small and large intestines (Figures 1F–1P; Figure S1A available online). Endoscopically and histologically, villi in the small intestine (SI) had a thickened and deformed appearance and were often branched (Figures 1I–1L). Changes in the large intestine (LI) included crypt hyperplasia and mucosal protrusions resembling villous structures that replaced the normal crypt pattern (Figures 1M–1P). This generalized hyperplasia was characterized by focal serrated epithelial formations, which had cytomorphologic features of human microvesicular or goblet cell-rich hyperplastic (serrated) polyps (Figures 1Q–1T and S1B). Both types were present in the LI, whereas microvesicular hyperplasia was predominant in the SI. Because of this resemblance to human serrated hyperplasia (Figure S1B), we refer to the histology in the mouse as murine serrated hyperplasia (mSH).

Like in human serrated hyperplastic polyps, there was a mild increase in the number of proliferating cells in mSH as compared to wild-type mucosa (Figures S1C and S1D). Ki67-positive cells were present in the mid and/or upper crypt in *Vil-Cre;Braf* ^*LSL-V637E*/+^ intestines but were restricted to the lower crypt in wild-type intestines (Figure S1C). Hyperproliferation seems to be the underlying mechanism of the hyperplastic changes because apoptosis was not reduced in *Vil-Cre;Braf*^*LSL-V637E*/+^ intestines as compared to wild-type mucosa (Figures S1E and S1F). We also intercrossed *LSL-Braf*^*V600E*^ mice with *Lgr5-EGFP-IRES-CreERT2* knockin mice. Tamoxifen-inducible Lgr5-Cre allowed stochastic activation of mutant Braf in a part of the intestinal stem cells, thereby inducing hyperplastic polyps in nonhyperplastic surrounding mucosa (Figure S1G). *BRAF* mutations have been observed in human serrated polyps occurring sporadically or in serrated polyposis syndrome and we show here that BRAF^V600E^ is indeed the underlying initiating event that is sufficient to induce lifelong sustained hyperplasia.

### BRAF^V600E^ Induced Serrated Tumorigenesis Progresses through a Hyperplasia/Adenoma/Carcinoma Sequence

**Figure 2:**
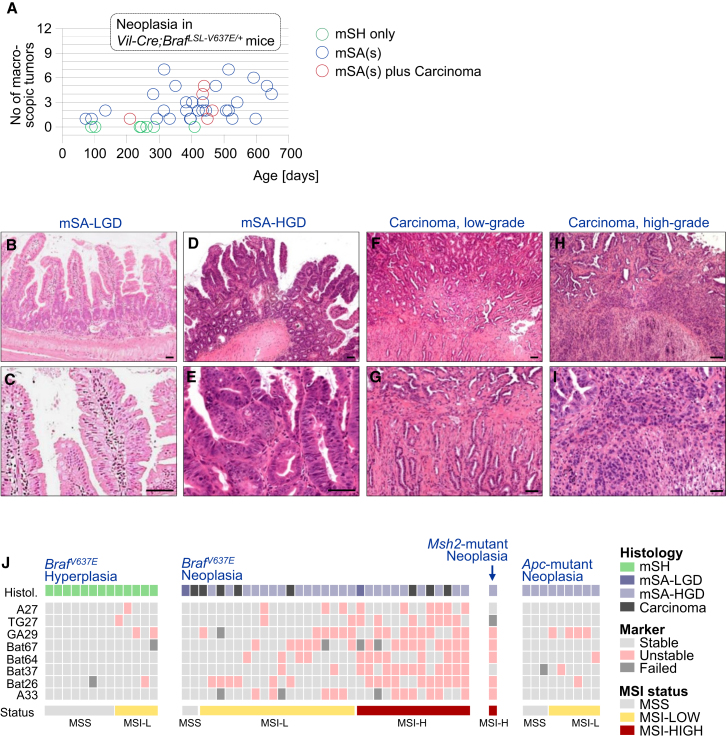
Intestinal Neoplasia Development in *Vil-Cre;Braf*^*LSL-V637E*/+^Mice (A) Overview of intestinal neoplasia development in *Vil-Cre;Braf*^*LSL-V637E*/+^mice. Each circle represents one mouse. Green circles, mice without macroscopic neoplasia. Blue circles, mice with macroscopic serrated adenomas mSAs (defined as tumors > 2 mm with dysplasia, identified at necropsy). The mSA number is indicated on the *y* axis. Animals represented by red circles had mSA(s) plus at least one carcinoma. Microscopic dysplasia is not shown. (B–I) Serrated dysplasia and adenocarcinoma in *Vil-Cre;Braf*^*LSL-V637E*/+^mice. Scale bars, 50 μm. Murine serrated adenoma with low-grade dysplasia (mSA-LGD) in the small intestine showing tubulovillous architecture and serrated, eosinophilic adenomatous epithelium (B and C). Murine serrated adenoma with high-grade dysplasia (mSA-HGD), showing tubulovillous architecture, sparse serration, and a high degree of atypia (D and E). Low-grade adenocarcinoma, showing predominant tubular differentiation (F and G). High-grade adenocarcinoma with remnants of tubular structures in the upper left part, but predominant loss of tubular differentiation in other areas (H and I). (J) Microsatellite instability in Braf^V637E^-induced hyperplasia/neoplasia as well as *Apc*- and *Msh2* mutant tumors. Eight markers were used for MSI-typing (see the Supplemental Experimental Procedures). Each column represents one sample. Samples were defined as microsatellite stable (MSS; all eight markers stable), MSI-Low (MSI-L; one or more, but < 40% of markers unstable) or MSI-H (≥40% of markers unstable). See also Figure S2 and Table S1.

To investigate whether mSH progresses to dysplasia, we aged *Vil-Cre;Braf*^*LSL-V637E*/+^mice up to 18 months and sacrificed them at various time points. Hyperplasia to dysplasia progression was often observed at a young age (2–3 months), at which time some animals already developed macroscopic tumors (>2 mm) with dysplasia. At 10 months, virtually all mice had such dysplastic lesions, often large numbers (Figure 2A). Histologically, Braf^V637E^-induced dysplastic lesions had features of human traditional serrated adenomas (TSAs), including crypt elongation and a serrated eosinophilic adenomatous epithelium (Figures 2B–2E and S1B). Although both TSAs and SSAs are associated with mutant *BRAF* in humans, we did not observe SSA in our model. A possible reason is that mouse tumors were predominantly in the SI (only five of 95 tumors were in the large intestine), where the specific morphologic features of human colonic SSAs might not develop. To avoid misleading nomenclature by drawing inadequate morphologic parallels between murine SI lesions and human LI tumors, we refer to dysplastic lesions as “murine serrated adenoma with dysplasia” (mSA) or more specifically as mSA with low-grade dysplasia (mSA-LGD) or high-grade dysplasia (mSA-HGD).

Macroscopically, Braf^V6*37*E^-induced neoplasia resembled human *BRAF* mutant colonic tumors, which frequently show a nonpolypoid sessile growth pattern (Figure S2A). Proliferation rates were increased on average 2.4-fold in mSA-LGD and 9.1-fold in mSA-HGD as compared to hyperplasia (Figures S2B–2D). Like human *BRAF* mutant tumors, mouse mSAs frequently showed abundant mucin production and stained positive for Alcian blue (Figure S2E).

In a subset of mice (n = 5) dysplasia progressed to invasive carcinomas: 8.3% (1/12) of *Vil-Cre;Braf*^*LSL-V637E*/+^ mice younger than 10 months and 13.8% (4/29) of mice older 10 months had cancers (Figure 2A). Two of these cancers were low-grade tumors (well and moderately differentiated), and three were high-grade cancers (poorly or undifferentiated; glandular structures in less than 50% of the tumor). Examples are shown in Figures 2F–2I. Across a larger set of Braf^V637E^-induced cancers in *p53* or *p16* mutant backgrounds (Table S1 and detailed below), we found that 30% of tumors were high grade. Collectively these results describe a mouse model of serrated intestinal cancer, which provides functional evidence for the key role of mutant *Braf* in tumor initiation.

### Braf^V637E^-Induced Murine Intestinal Tumors Are Frequently Microsatellite-Unstable

High level microsatellite instability (MSI-H) occurs in 50% of human *BRAF* mutant cancers (Rajagopalan et al., 2002). It is however not understood at which stage MSI develops and whether *BRAF* mutations are cause or consequence of MSI. To address this question, we assessed the MSI status in Braf^V6*37*E^-induced serrated hyperplasia and neoplasia as well as in *Msh2*^−*/*−^ and *Apc*^*min*^ control tumors. A panel of eight microsatellite repeats was used for MSI typing (Figure 2J; Supplemental Experimental Procedures). We found that all Braf^V6*37*E^-induced hyperplastic polyps (13/13) were microsatellite stable (MSS) or MSI-low (MSI-L). Contrarily, 39.4% (13/33) of Braf^V6*37*E^-induced mSAs and carcinomas were MSI-H and only 6% (2/32) were MSS (Figure 2J). MSI-H was observed at similar frequencies in mSAs (10/25; 40%) and carcinomas (3/8; 37.5%). *Apc*^*min*^-induced adenomas were all (9/9; 100%) MSS or MSI-L. The lack of MSI-H in mSH, but its presence in all subsequent stages of tumorigenesis (mSA-LGD, mSA-HGD and carcinoma) suggests its early development during Braf^V6*37*E^-initiated transformation.

### P53 Tumor Suppression Inhibits Invasion and Metastasis but Does Not Affect Tumor Initiation in Braf^*V637E*^-Induced Tumorigenesis

The long latency and low penetrance of cancer development might be explained by the ability of constitutive MAPK signaling to activate anti-oncogenic programs, most notably the *p16*^*INK4a*^/Rb and *p19*^*ARF*^/p53 pathways (Palmero et al., 1998; Lin et al., 1998).

**Figure 3:**
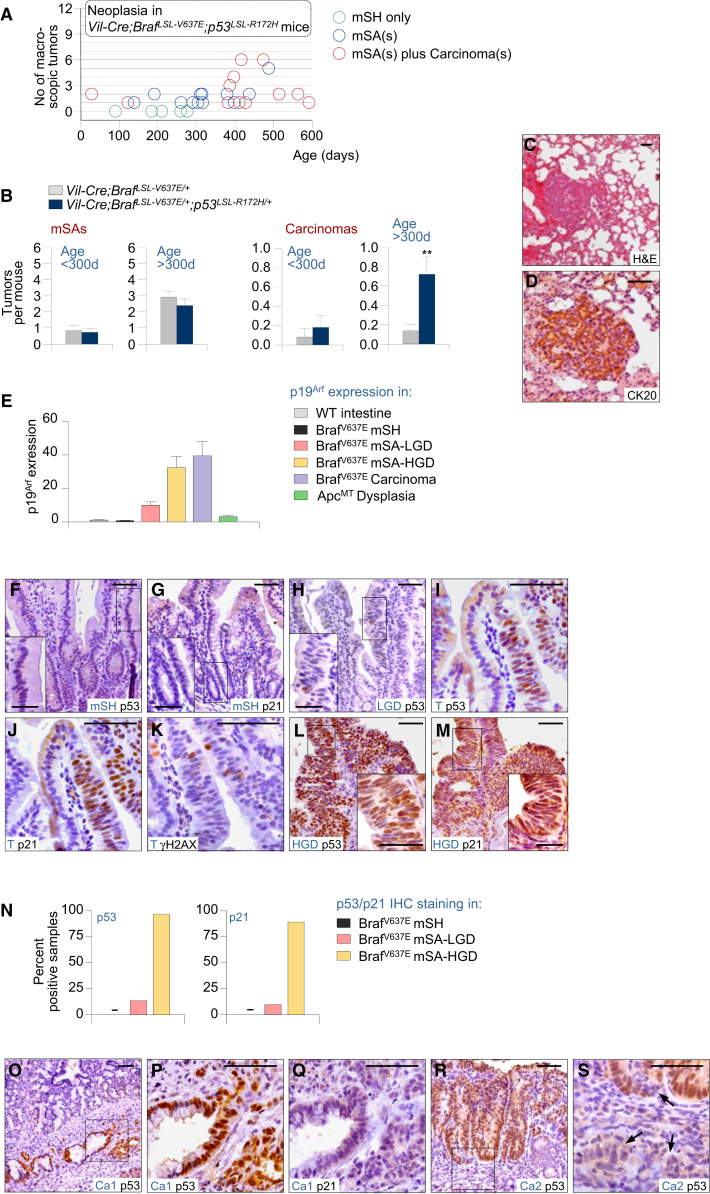
P53 Is Activated Late during Braf^V637E^-Induced Intestinal Tumorigenesis and Plays an Important Role in Invasiveness Control (A) Intestinal tumor type, number, and latency in *Vil-Cre;Braf*^*LSL-V637E*/+^*;p53*^*LSL-R172H/+*^ mice. Each circle represents one mouse. Green circles, mice without macroscopic neoplasia. Blue circles, mice with macroscopic serrated adenomas mSAs (defined as tumors > 2 mm with dysplasia, identified at necropsy). (B) Average adenoma and carcinoma number in *Vil-Cre;Braf*^*LSL-V637E*/+^*;p53*^*LSL-R172H/+*^ mice as compared to *Vil-Cre;Braf*^*LSL-V637E*/+^ animals. ^∗∗^p < 0.01, Mann-Whitney rank sum test. Error bars, SEM. (C and D) Lung metastasis of intestinal cancer in a *Vil-Cre;Braf*^*LSL-V637E*/+^*;p53*^*LSL-R172H/+*^ mouse stains positive for the enterocytes-specific marker CK20. Scale bars, 50 μm. (E) Expression of p19^Arf^ in small intestinal samples with the indicated genotypes and histology; n = 55 (total); error bars, SEM p19^Arf^ was normalized to *Actb* expression. mSH, murine serrated hyperplasia; mSA-LGD, murine serrated adenoma with low-grade dypslasia; mSA-HGD, murine serrated adenoma with high-grade dysplasia. Error bars, SEM. (F–M) p53, p21, or γH2AX staining in SI samples from *Vil-Cre;Braf*^*LSL-V637E*/+^ mice: hyperplasia (F and G), low-grade dysplasia (H), area with hyperplasia and dysplasia (I–K), high-grade dysplasia (L and M). T, transition hyperplasia/dysplasia; Scale bars, 50 μm for micrographs, 20 μm for insets. (N) Frequency of positive staining for p53 and p21 in indicated sample types from *Vil-Cre;Braf*^*V637E/+*^ mice. N = 110 (for p53); n = 35 (for p21). (O–S) p53 and p21 staining in two carcinomas from *Vil-Cre;Braf*^*LSL-V637E*/+^ mice. A cancer with spontaneous *p53* mutation (S152R) stains positive for p53 but negative for p21 (Q and R). In a second cancer there was loss of p53 expression in invading cancer cells (arrows) but not in the area of dysplasia (R and S). Ca, carcinoma. Scale bars, 50 μm. See also Tables S2 and S3.

To investigate the role of *p53* in Braf^V6*37*E^-induced intestinal tumorigenesis, we used *p53*^*LSL-R172H/+*^ knockin mice, expressing the equivalent of the dominant-negative human *TP53*^*R175H*^ conditionally (Olive et al., 2004). We intercrossed them with *Vil-Cre;Braf*^*LSL-V637E*/+^ mice, aged the different double- and triple-transgenic cohorts, and monitored mice for tumor development (Figure 3A). We found that the average number of mSAs per mouse was similar in *Vil-Cre;Braf*^*LSL-V637E*/+^ and *Vil-Cre;Braf*^*LSL-V637E*/+^*;p53*^*LSL-R172H/+*^ animals (2.3 and 1.8, respectively; Figure 3B; Table S2). Likewise, the proportion of mice developing mSAs did not differ between groups (82.9% and 82.8%, respectively, Table S2), suggesting that the p53 pathway does not restrain dysplasia initiation.

In sharp contrast, invasive cancers were found considerably more frequently in *Vil-Cre;Braf*^*V637E*/+^*;p53*^*LSL-R172H/+*^ mice (Figure 3B; Table S3). Fifty-six percent of *Vil-Cre;Braf*^*LSL-V637E*/+^*; p53*^*LSL-R172H/+*^ animals at an age of 10–20 months had carcinomas, as compared to 13.8% of mice in the *Vil-Cre;Braf*^*LSL-V637E*/+^ cohort (p = 0.002, χ^2^ test). The average number of cancers was 5.2 times higher in the *Vil-Cre;Braf*^*LSL-V637E*/+^*;p53*^*LSL-R172H/+*^ cohort (p = 0.007; Mann-Whitney rank sum test). Some animals had more than one synchronous cancer and 25% (3/12) of mice with cancer had metastases to local lymph nodes, pancreas, or lungs (Figures 3C and 3D). All together, these data show that p53 does not affect early stages of Braf^V6*37*E^-induced tumorigenesis but plays an important role in invasiveness control.

### Activation of p53 Tumor Suppression during Advanced, but Not Early Tumorigenesis

We next examined at which stage of tumorigenesis the p53 pathway becomes activated (Figures 3E–3N). We performed immunohistochemistry for p53 and its target gene p21 in wild-type as well as *Braf* mutant hyperplasia and neoplasia. Immunoreactivity for p53 was negative in all wild-type intestines (n = 21), all Braf^V637E^-induced mSHs (n = 43), and most mSAs-LGD (Figures 3F–3H and 3N). Only 5/37 mSAs-LGD were p53-positive (example in Figure 3I). We detected however marked p53 expression in 97% (28/29) of mSAs-HGD (Figures 3L and 3N). There was a strong concordance of p53 and p21 immunoreactivity in all samples. Similar to p53, p21 IHC was negative in all wild-type intestines (n = 21), all Braf^V637E^-induced mSHs (n = 15), and most (10/11) mSAs-LGD but was present in the majority (8/9; 89%) of mSAs-HGD (Figures 3G, 3J, 3M, and 3N). These data suggest selective activation of p53 tumor suppression during advanced but not early stages of tumor evolution.

To investigate the mechanism of p53 activation, we first stained for the DNA damage marker γH2AX. Oncogene-induced DNA damage can activate p53 via ARF-independent pathways (Sherr and McCormick, 2002). All mSHs (n = 20) or mSAs-LGD (n = 12) were γH2AX-negative (Figure 3K), and only three of 17 mSAs-HGD (all p53/p21-positive) showed evidence for activation of the DNA damage response. In contrast, p19^Arf^ expression increased substantially during tumor progression: average normalized p19^Arf^ mRNA levels were similar in Braf^V6*37*E^-induced mSHs (0.7) and wild-type mucosa (1.0), but were increased 9.9-, 32.3-, and 39.4-fold in mSAs-LGD, mSAs-HGD, and carcinomas, respectively (Figure 3E). We conclude that p53 is activated mainly via p19^Arf^ in advanced Braf^V637E^-iduced tumorigenesis, explaining its late stage specific function.

### Selective Pressure for p53 Inactivation Develops at Advanced Stages of Tumor Evolution

To examine whether *p53* mutations occur spontaneously during Braf^V637E^-induced intestinal tumorigenesis, we next sequenced *p53* in *Braf* mutant tumors. Whereas mSAs (n = 17) did not have *p53* mutations, we identified a missense mutation (S152R; equivalent of human T155A) in one of the two carcinomas. S152R leads to stabilization of nonfunctional p53, as evidenced by loss of p21 expression in cancer cells (Figures 3O–3Q). In the second cancer, p53 expression was lost whereas the surrounding dysplasia, which gave rise to the cancer, was p53-positive (Figures 3R and 3S). These results suggest late stage specific selective pressure to inactivate p53, further supporting the importance of p53 for invasiveness control.

### Inactivation of p16 Promotes Advanced Phases of Braf^V637E^-Induced Intestinal Tumorigenesis

**Figure 4:**
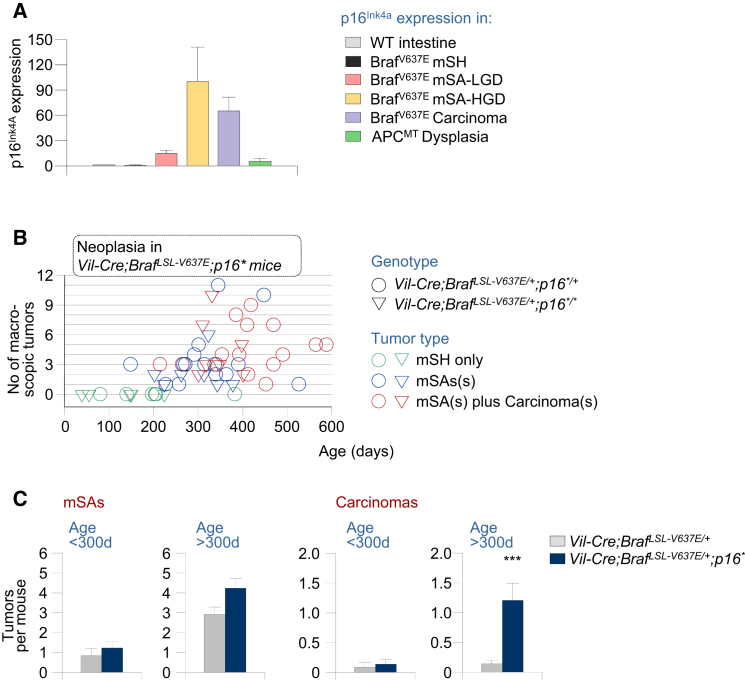
The Role of p16-Dependent Tumor Suppression in Braf^V637E^-Induced Intestinal Carcinogenesis (A) p16^Ink4a^ expression (qRT-PCR; normalized to *Gapdh*) in SI samples with the indicated genotypes and histology; n = 55 (total); Error bars, SEM. (B) Intestinal tumor type, number, and latency in *Vil-Cre;Braf*^*LSL-V637E*/+^*;p16^∗^*^*/+*^ and *Vil-Cre;Braf*^*LSL-V637E*/+^*;p16^∗^*^*/*^*^∗^* mice. Each circle/triangle represents one mouse. Green circles/triangles, mice without macroscopic neoplasia. Blue circles/triangles, mice with macroscopic serrated adenomas mSAs (defined as tumors > 2 mm with dysplasia, identified at necropsy). Note that some mice had multiple independent cancers. (C) Average mSA and carcinoma number in *Vil-Cre;Braf*^*LSL-V637E*/+^*;p16^∗^* mice as compared to *Vil-Cre;Braf*^*LSL-V637E*/+^ animals. *p16^∗^* indicates all p16 mutant mice (hetero- and homozygous); ^∗^p < 0.001, Mann-Whitney rank sum test. Error bars, SEM. See also Figure S3 and Tables S4 and S5.

To examine the role of *p16*^*Ink4a*^, we first analyzed *p16*^*Ink4a*^ expression in *Braf* mutant healthy and neoplastic intestines (Figure 4A). Whereas *p16*^*Ink4a*^ expression was similar in *Braf* mutant mSH and WT mucosa, there was a marked upregulation of *p16*^*Ink4a*^ expression in *Braf* mutant neoplasia. This effect was less pronounced in mSAs-LGD than in mSAs-HGD, in which *p16*^*Ink4a*^ was induced on average 100-fold (Figure 4A). Thus, similarly to Braf^V6*37*E^-induced Arf/p53 activation, substantial p16^Ink4a^ activation is only triggered at advanced stages of tumorigenesis. This is consistent with observations in humans, where *p16* was upregulated in *BRAF* mutant premalignant lesions (SSAs and TSAs) but not in hyperplasia (Kriegl et al., 2011).

To investigate whether *p16*^*Ink4a*^ inactivation occurs spontaneously in Braf^V6*37*E^-induced tumors, we performed comparative genomic hybridization, sequencing, and methylation analysis of the *cdkn2a* locus. We did not identify *Cdkn2a* mutations or copy number alterations in any of the 12 TSAs and eight carcinomas analyzed (data not shown). In a subset of Braf^V637E^-induced mSAs-HGD and carcinomas, however, we found partial CpG island methylation in the *p16*^*Ink4a*^ (but not *p19*^*Arf*^) promoter (Figure S3), similar to observations in humans (Kriegl et al., 2011).

To study the effect of *p16*^*Ink4a*^ inactivation in vivo, we used *p16*^*Ink4a^∗^*^ mice, which have a point mutation that is silent in the *p19*^*Arf*^ reading frame but introduces a stop codon in *p16*^*Ink4a*^ (Krimpenfort et al., 2001). *Vil-Cre;Braf*^*LSL-V637E*/+^ mice with heterozygous or homozygous mutation of *p16*^*Ink4a*^ *(Vil-Cre;Braf*^*LSL-V637E*/+^*;p16*^*Ink4a^∗^*^*)* were aged and sacrificed at different time points to assess tumor incidence and latency (Figure 4B). We observed 1.3-fold increased numbers of mSAs in *Vil-Cre;Braf*^*LSL-V637E*/+^*;p16*^*Ink4a^∗^*^ animals as compared to *Vil-Cre;Braf*^*LSL-V637E*/+^ mice, but this was statistically not significant (Figure 4C; Table S4). In contrast, carcinoma development was significantly increased in *Vil-Cre;Braf*^*LSL-V637E*/+^*;p16*^*Ink4^∗^*^ mice, which had on average 6.4 times as many cancers as *Vil-Cre;Braf*^*LSL-V637E*/+^*;p16*^*Ink4a+/+*^ mice (p < 0.001; Figure 4C; Table S5). Many of the mice developed multiple synchronous carcinomas and, in some animals (3/24), these tumors were metastatic. All together, these results show that Arf/p53 and p16 exert independent critical tumor-suppressive effects, which are mainly operative at advanced stages of Braf^V637E^-induced intestinal tumorigenesis.

### Intensification of Map Kinase Signaling during Dysplasia Progression

**Figure 5:**
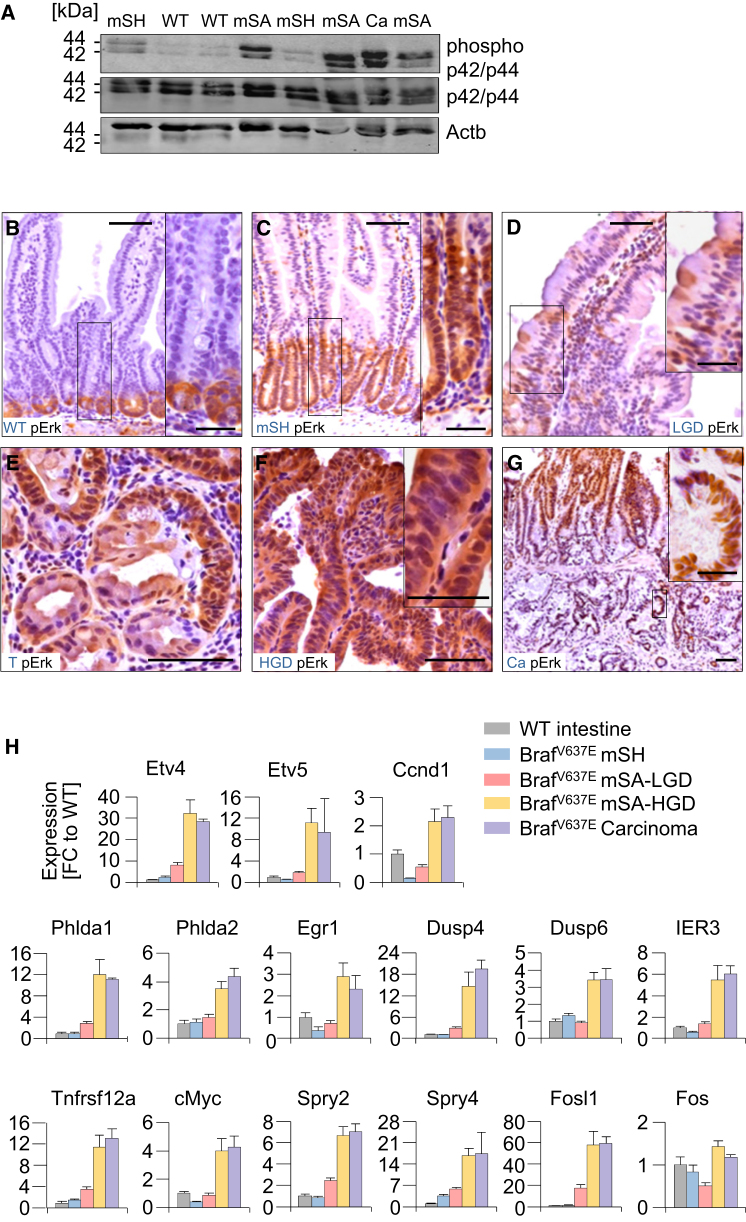
Dysplasia Progression Is Associated with Stage-Specific MAPK Signaling Amplification (A–H) Map-kinase pathway activation and signaling output at different stages of Braf^V637E^-induced tumor development. Phospho Erk1/2 (phospho-p42/p44) and Erk1/2 (p42/p44) western blot in indicated samples (A). Phospho-Erk IHC staining in indicated samples (B–G). Note in (E) the pErk positive/negative cells in dysplastic/nondysplastic areas of one gland. mSH, murine serrated hyperplasia; LGD, low-grade dypslasia; T, transition (hyperplasia/dysplasia); HGD, high-grade dysplasia; Ca, carcinoma. Scale bars, 50 μm for micrographs, 20 μm for insets. (H) Expression of Erk target genes (qRT-PCR; normalized to *Gapdh*) in SI samples with the indicated histology and genotypes. FC, fold-change compared to wild-type intestine; n = 41; Error bars, SEM. See also Figure S4.

Because Braf-induced Mapk signaling does not seem to engage intrinsic tumor suppression in early tumorigenesis, we next assessed the MAPK pathway activity at different stages of tumor evolution. Unexpectedly, phospho-p42/p44 MAPK (pErk) protein levels were only slightly increased in Braf^V6*37*E^-induced mSH as compared to wild-type mucosa but were highly induced in mSAs and carcinomas (Figure 5A). Immunohistochemistry revealed that in wild-type mucosa and Braf^V6*37*E^-induced mSH, pErk reactivity was mostly confined to the lower parts of the crypts (Figures 5B and 5C). In mSAs-LGD, few scattered pERK-positive cells were occasionally additionally detected in dysplastic areas (Figure 5D). mSAs-HGD and carcinomas, however, stained uniformly positive for pErk (Figures 5E–5G). Compared to wild-type mucosa, the number of pERK-positive cells per gland was increased 1.4-, 2.4-, and 6.6-fold in mSH, mSAs-LGD, and mSAs-HGD, respectively (Figure S4A). To assess the functional relevance of these observations, we examined expression of a panel of 15 Erk target genes (Pratilas et al., 2009) using qRT-PCR (Figure 5H) or immunohistochemistry (Figure S4). The panel of markers includes a number of effectors of Ras/Raf-induced transformation, such as the ETS family members *Etv4* and *Etv5* or *cMyc* and *Ccnd1*, and genes involved in the feedback regulation of Mek/Erk signaling, such as *Dusp4*, *Dusp6*, *Spry2*, and *Spry4*. We found that the transcriptional output of the Erk pathway was only slightly induced in Braf^V6*37*E^-dependent mSH and mSAs-LGD (average fold-change across target genes: 1.1 and 3.4, respectively) but was strongly upregulated in mSAs-HGD and carcinomas (average fold-change across target genes: 13.0 and 12.6, respectively). The extent of induction varied between markers and was highest for Fosl1 (60-fold induction in Braf^V6*37*E^-induced mSAs-HGD).

### Wnt Pathway Activation during Dysplasia Progression

**Figure 6:**
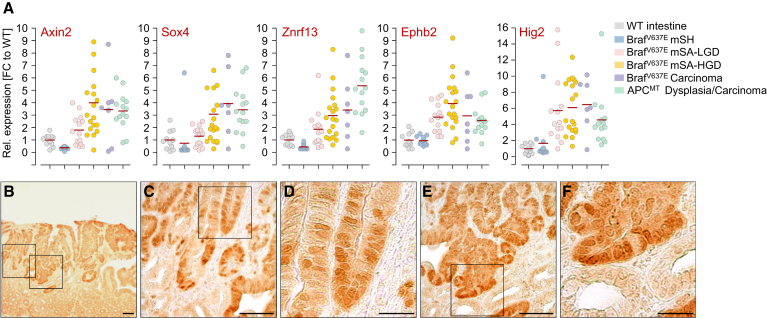
Frequent Wnt Pathway Activation during Braf^V637E^-Induced Dysplasia Progression (A) Expression of Wnt target genes (qRT-PCR; normalized to *Gapdh*) in SI samples with indicated histology and genotypes. n = 11 (wild-type), 53 (*Braf* mutant), and 14 (*Apc* mutant tissues). Red lines, mean. (B–F) β-Catenin staining in Braf^V6*37*E^-induced hyperplasia/dysplasia. Scale bars, 50 μm (B, C, and E) or 20 μm (D and F). See also Figure S5 and Table S6.

To examine the role of the Wnt pathway in Braf^V6*37*E^-induced tumorigenesis, we first analyzed the expression of ten different Wnt target genes in a total of 78 samples (Figures 6A and S5). We found that Wnt target gene expression was similar in wild-type mucosa and Braf^V6*37*E^-induced mSH but was upregulated in a large number of Braf^V6*37*E^-induced mSAs-HGD (and occasionally in mSAs-LGD) to similar levels as in *Apc*^*min*^-induced tumors. Immunohistochemical staining of beta-catenin (*Ctnnb1*), a key effector of Wnt pathway activation, was performed to further confirm these observations. As in wild-type mucosa, there was no evidence for nuclear β-catenin accumulation in mSHs (n = 42) and the majority of mSAs-LGD (14/15). In contrast, there was diffuse or focal nuclear β-catenin accumulation in a substantial part of mSAs-HGD (8/14) and carcinomas (2/4) (Figures 6B–6F).

To analyze the mechanisms of Wnt pathway activation, we performed whole-exome sequencing of 20 *Braf* mutant tumors. We identified a number of mutations in known Wnt pathway genes (Table S6), including intracellular components of the Wnt pathway (e.g., *Apc*, *Ctnnb1*, *Gsk3b*, and *Axin2*), Wnt receptors (e.g., *Lrp8* and *Fzd9*), or negative regulators of Wnt signaling (e.g., *Lrp1b* and *Lrp4*). We then further analyzed the most frequently altered genes *(Apc, Ctnnb1*, and *Lrp1b)* in another 46 tumors and found mutations in these three genes in 21/66 samples: *Apc* (n = 6), *Ctnnb1* (n = 9), and *Lrp1b* (n = 6). Wnt pathway mutations frequently occurred in high-grade dysplasia, suggesting an early requirement during tumorigenesis. Only missense, nonsense, essential splice site mutations or frameshift-causing indels were observed (no silent mutations), suggesting a strong enrichment for functionally relevant events. For example, *Apc* mutations were mostly nonsense or frameshift mutations, whereas *Ctnnb1* mutations were recurrent activating mutations at specific positions that have also been described in humans (e.g., T141I). Missense mutations in *Lrp1b*, a negative regulator of Wnt signaling, have been found earlier in *Braf* mutant human melanoma (Nikolaev et al., 2012). All together, these results provide strong evidence for an important role of Wnt pathway activation during early dysplasia progression. It is worth noting that in some tumors with strong Wnt target gene expression, no mutations in Wnt pathway genes were found, suggesting additional unidentified mechanisms.

### Large-Scale Drug Screening Identifies Targetable Nodes in Braf-Induced Tumorigenesis

**Figure 7:**
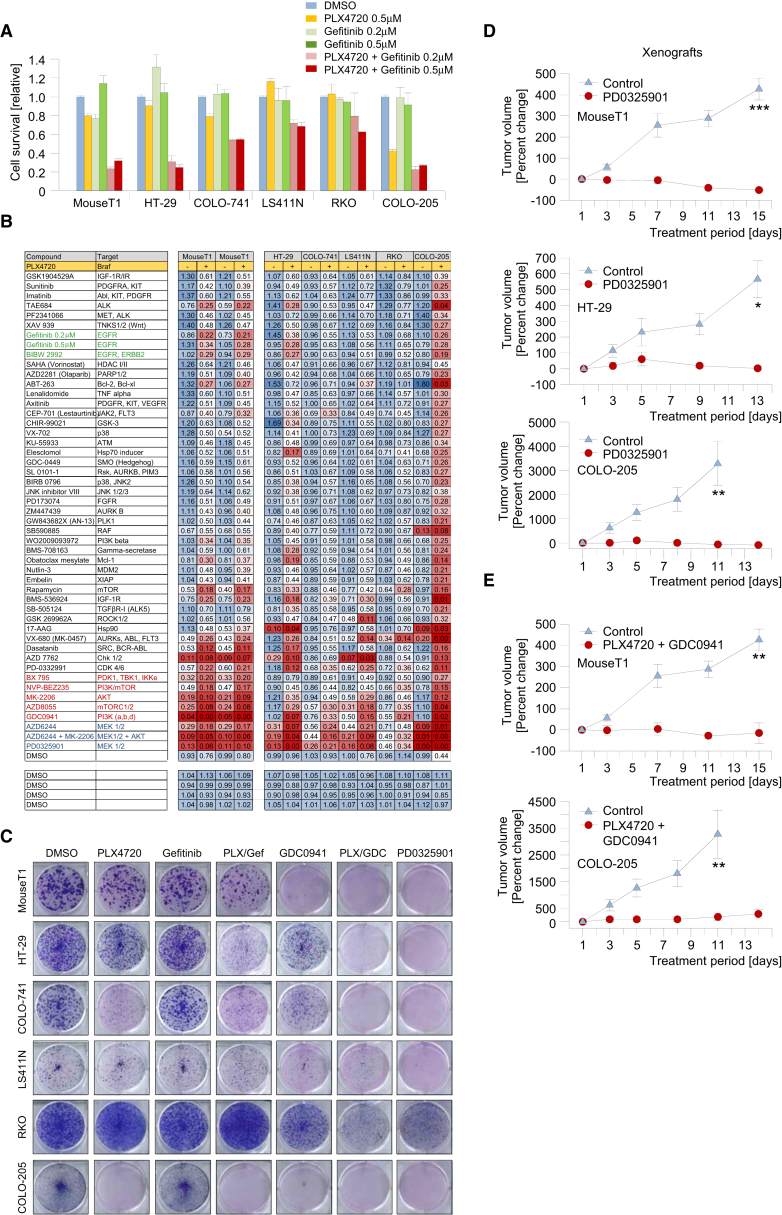
Systematic Drug Screening Identifies Targets for Therapeutic Intervention in Braf^V637E^-Induced Murine and Human Cancers (A) In vitro growth inhibition assays (CellTiter-Blue assay) using a murine and five human *BRAF* mutant intestinal cancer cell lines. Drug treatment was performed for 6 days. Error bars, SEM; n = 2. (B) Systematic drug sensitivity screens using 50 compounds. For each cell line, drugs were used as single agents (left columns) or in combination with 0.5 μM PLX4720 (right columns). Cell viability was determined after 6 days of treatment using CellTiter-Blue. Results are shown relative to DMSO control treatment. One of two determinations with similar results is shown. (C) Long-term colony-forming assays. Cells were treated with PLX4720 (0.5 μM), Gefitinib (0.5 μM), the PI3K inhibitor GDC0941 (0.5 μM), and the Mek inhibitor PD0325901 (0.002 μM) as single agents or their combination as indicated. One of two determinations with similar results is shown. (D) The Mek inhibitor PD0325901 suppresses tumor growth in allo- and xenograft models. The *Braf* mutant murine (MouseT1) and human (HT-29 and COLO-205) cell lines were transplanted s.c. into Nod Scid IL12Rg^null^ (NSG) mice. Treatment was started when tumors were palpable (day 1). Animals were treated once daily with vehicle (control) or 25 mg/kg/day of PD0325901 for 15 days by oral gavage. ^∗^p < 0.05; ^∗∗^p < 0.01; ^∗∗∗^p < 0.001; Mann-Whitney rank sum or t test; Error bars, SEM; n = 4–5 mice per group. (E) Combinatorial Braf/PI3K inhibition suppresses tumor growth of MouseT1 and COLO-205 cells, transplanted s.c. into NSG mice. Animals were treated with vehicle (control) or PLX4720 50 mg/kg/day once daily plus GDC09041 75 mg/kg/day twice daily by oral gavage. Error bars, SEM; n = 3–5 mice per group; ^∗∗^p < 0.01; t test. See also Figure S6 and Table S7.

To test the sensitivity of *Braf*^*LSL-V637E*/+^-induced intestinal cancer cell lines to Braf inhibition we performed short-term proliferation assays. Overall, only minor growth inhibition was observed for *Braf* mutant mouse and human colorectal cancer cell lines treated with 5 μM PLX4720, a selective inhibitor of mutant Braf (Figures 7A and 7B).

Braf inhibition was proposed to cause feedback activation of the epidermal growth factor receptor (EGFR) in human *BRAF* mutant CRCs (Prahallad et al., 2012). We therefore treated mouse and human *BRAF* mutant cell lines with the EGFR small molecule kinase inhibitor, Gefitinib, alone or in combination with PLX4720. As expected, Gefitinib and PLX4720 synergized in-growth inhibition (Figures 7A and 7B). The murine intestinal cancer cell line MouseT1 (from a *Vil-Cre;Braf*^*LSL-V637E*/+^*;p53*^*LSL-R172H/+*^ mouse), had similar sensitivity to combinatorial PLX4720/Gefitinib treatment as HT-29 (WiDr), one of the three human cell lines tested by Prahallad and colleagues (Figures 7A and 7B; inhibition of proliferation by 60%–70%). It seems, however, that the effectiveness of PLX4720/Gefitinib varies considerably among human cancers: in three of five tested human cell lines the effects were rather modest (growth inhibition by 25%–40%; Figure 7A and data not shown). We next performed long-term (14 days) clonogenic assays and again found that although PLX4720 and Gefitinib synergized in-growth inhibition, most of the treated cell lines retained variable levels of colony-forming capacity (Figure 7C).

To identify alternative drugs with effectiveness across cell lines, we performed high-throughput drug screening. We tested a large set of compounds inhibiting a broad range of molecules, pathways, and biologic processes (Figure 7B; Table S7). All compounds were tested alone or in combination with PLX4720 and for each cell line we performed 100 different short-term (6 day) sensitivity assays. These screens revealed several treatment approaches that were highly effective (Figure 7B). PD0325901, a Mek inhibitor, was the most effective single compound across cell lines in the short-term assays (Figure 7B). In the long-term clonogenic assay, it induced complete inhibition of colony-forming capacity in five of six cell lines and partial inhibition in the remaining line RKO (Figure 7C). The PI3K inhibitor GDC0941 was not effective as a single agent, but induced potent inhibition in combination with PLX4720 across cell lines (Figures 7B and 7C).

Some other drug combinations strongly inhibited selected cell lines, although they were not broadly effective across tumors. For example, the combination of PLX4720 plus the kinase inhibitor VX-680 was the most potent drug combination for the treatment of RKO, a highly resistant cell line to most other drugs. This shows the power of systematic drug screening to identify patient-specific treatment approaches even for highly resistant tumors. Another example is the combination of the Chk1/2 inhibitor AZD-7762 plus PLX4720, which was very effective in MouseT1, HT-29, LS411N, and COLO-205 and could potentially be a broadly effective alternative first-line or second-line combination.

### In Vivo Validation of Mek and Combinatorial Braf/PI3K Inhibition

To study the effectiveness of broadly effective drug combinations in vivo, we first transplanted mouse and human cell lines subcutaneously (s.c.) into immunodeficient Nod Scid IL12R-gamma null (NSG) mice and assessed their response to PD0325901. Treatment was started 7–14 days after s.c. injection of cells as soon as tumors were palpable. Animals were given PD0325901 or vehicle by oral gavage for 13–15 days. PD0325901 was highly effective, causing regression of tumors from all tested cell lines (Figure 7D). Figures S6A and S6B show that after 13–15 days of PD0325901 treatment there was complete inhibition of ERK phosphorylation in surviving tumor cells and that only very few scattered Ki67-positive cancer cells could still be observed in the necrotic tumor mass.

We next performed orthotopic transplantation of mouse and human *Braf* mutant cancer cell lines into the cecum of NSG mice. Fourteen days later, treatment was started with either vehicle or PD0325901. Mice were sacrificed after 17 days of treatment. Figures S6C–S6E show that vehicle-treated mice developed large tumors, which metastasized to local lymph nodes and the peritoneum, causing hemorrhagic ascites. In contrast, in the PD0325901-treated group, tumors were either not detectable or small (maximum 0.01 cm^3^).

To examine the effect of PD0325901 on proliferation in endogenous Braf^V637E^-induced tumors, we performed short-term treatments (5 days) of *Vil-Cre;Braf*^*LSL-V637E*/+^ mice. We used animals that were more than 1 year of age and were expected to have tumors. Figures S6F and S6G show that Ki67 immunoreactivity was weak in the majority of dysplastic cells in PD0325901-treated mice but was strong in tumors of vehicle-treated animals. All together, these data show that Mek inhibition is effective in the treatment of Braf-induced intestinal tumors in vivo.

To examine the effectiveness of combinatorial Braf/PI3K inhibition in vivo we treated s.c. transplanted murine and human cell lines with a combination of PLX4720 and GDC0941 or vehicle. Figures 7E, S6H, and S6I show that combined Braf/PI3K inhibition elicited potent growth inhibition in both models. Immunohistochemical staining revealed that proliferation was substantially inhibited in the PLX4720/GDC0941-treated group, with only few Ki67-positive cells being detectable in regressed tumor masses (Figure S6I). These data mirror the in vitro effectiveness of these treatments in vivo.

## Discussion

**Figure 8:**
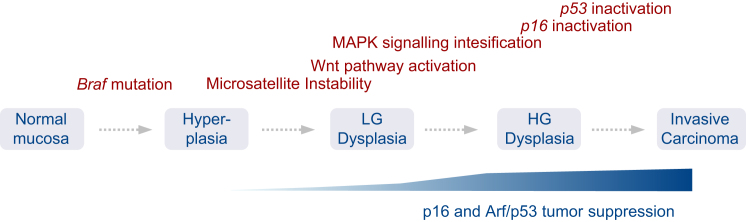
Progression Model of Braf^V637E^-Induced Intestinal Cancer Development *Braf* mutation induces sustained hyperplasia. MSI-H develops in 40% of cases and is observed in all subsequent stages of tumorigenesis, suggesting its early development. Dysplasia progression is driven by stage-specific Wnt pathway activation and Braf/Mek/Erk signaling intensification. Selective pressure for inactivation of the p16/Rb and Arf/p53 pathways develops late during tumorigenesis and promotes invasion and metastasis but does not accelerate early adenoma initiation. This late-stage specificity results from the inability of low-dose Mapk signaling to activate these tumor suppressors at early stages of tumorigenesis.

CRC is the second most common cancer in the Western world (Jemal et al., 2010). *BRAF* mutations occur in more than 10% of cases and define a subset that has a higher incidence than many other solid or hematopoietic tumor entities. Despite this high incidence, the molecular evolution of the disease is poorly understood. Since the discovery of *BRAF* mutations in colorectal cancer (Davies et al., 2002), a vast body of literature has been published on the association of *BRAF* mutations with other genetic and epigenetic events in CRC. However, the interpretation of these observations, their functional relevance, and the sequence of events driving tumorigenesis remained largely speculative. We established mouse models that recapitulate human *BRAF*^*V600E*^-associated intestinal pathology, including sustained hyperplasia, serrated adenomas and metastatic carcinomas. They reflect the macroscopic appearance of human *BRAF* mutant tumors (flat nonpolypoid neoplasia), their pathomorphologic characteristics (serrated and mucinous appearance), their genetic features (e.g., microsatellite instability), and their response to targeted therapeutics. Using these models, we dissected key aspects of the molecular evolution of these tumors. Our findings suggest a progression model of Braf^V637E^-induced carcinogenesis as summarized in Figure 8.

Engineered animal models that accurately recapitulate the characteristics of human disease are powerful tools for genetic and preclinical cancer research. Recently, *AhCre;Braf*^*LSL-V600E*/+^ mice have been used to examine the effects of mutant *Braf* in the intestine (Carragher et al., 2010), but intestinal tumorigenesis was difficult to study due to early onset Braf^V600E^-induced extraintestinal cancer development and lethality. This might explain why some of the main conclusions of that study are not supported by our data or by observations in human samples. For example, Braf-induced hyperplasia was described to be transient in that model but is sustained in human *BRAF* mutant hyperplastic polyps and in our model. Likewise, our data as well as work performed on human samples (Fujita et al., 2011; Yachida et al., 2009) refute that Braf^V6*37*E^ expression induces generalized Wnt pathway activation in human intestinal hyperplasia, as suggested in that study (Carragher et al., 2010).

Alterations in *APC* occur in 80% of human CRCs. Because *APC* mutations are less frequent in the *BRAF* mutant CRC subset, it was largely assumed that BRAF^V600E^-associated intestinal tumorigenesis is Wnt-independent (Samowitz et al., 2007; Jass, 2007). However recent studies, which used β-catenin immunohistochemistry rather than mutation analysis as a measure of Wnt pathway activation found nuclear β-catenin reactivity in a large part of BRAF mutant advanced (but not early) human adenomas and in carcinomas (Fujita et al., 2011; Yachida et al., 2009). Our results in mice not only mirror this situation but explain these apparent discrepancies and establish stage-specific Wnt pathway activation as a hallmark of Braf^V6*37*E^-induced dysplasia progression. We found a large spectrum of Wnt pathway genes mutated in our murine tumors, similarly to human “hypermutated” tumors, as described recently by the Cancer Genome Atlas research network (Muzni et al., 2012).

In humans, serrated colorectal cancers are associated with either *BRAF* or *KRAS* mutations. A series of studies in *Kras* mutant mouse models demonstrated *Kras*-initiated serrated tumorigenesis (Janssen et al., 2002; Haigis et al., 2008; Bennecke et al., 2010; Feng et al., 2011). The Braf^V637E^- and *Kras*^*G12D*^-induced pathways differ however in several aspects. First, *Braf*^*V637E*^ seems to be a highly potent oncogene, inducing cancers even on an otherwise wild-type background. The tumor incidence in the *Braf*^*V637E*^-model is even higher than in transgenic lines expressing multiple Kras^G12V^ copies (Janssen et al., 2002). Second, while Kras^G12D^-induced serrated tumors do not seem to require Wnt pathway activation (Bennecke et al., 2010), we find evidence for Wnt signaling induction in a substantial part of Braf^V637E^-induced high-grade tumors. Third, murine and human intestinal tumors with *KRAS* mutations are mostly MSI-stable or MSI-low (Noffsinger, 2009; Bennecke et al., 2010), whereas *BRAF* mutant human tumors are frequently MSI-high (Rajagopalan et al., 2002). This genetic feature is faithfully recapitulated in our model, which not only causally links Braf^V637E^ to MSI development, but also demonstrates its early stage development. Finally, *BRAF* mutant human tumors have—in contrast to *KRAS* mutant serrated tumors—a predilection for proximal (right-sided) location and are more frequent in females than in males (Spring et al., 2006). Collectively, these data provide compelling evidence for the existence of different pathways to serrated intestinal tumorigenesis.

Our studies revealed that p19^Arf^/p53 and p16^Ink4A^ exert independent critical tumor-suppressive effects. *p16*^*Ink4A*^ inactivation is a critical early event promoting neoplastic transformation in some types of cancers, whereas in other tumor types it has been described to be an intermediate or late event (Romagosa et al., 2011). A recent study even showed that extrinsic signals present in a emerging tumor induce local non-cell-autonomous *p16*^*Ink4A*^ expression (Burd et al., 2013). We found that experimental *p16*^*Ink4A*^ inactivation has only a mild effect on dysplasia initiation, but substantially increases the incidence of cancers. The lack of p16^Ink4A^ tumor suppression during early stages was surprising, given that p16^Ink4A^ was believed to mediate an early BRAF^V600E^-induced senescence program in nontransformed enterocytes. BRAF^V600E^-induced senescence has been extensively studied in cutaneous nevi, where it induces a near-total block of proliferation (Michaloglou et al., 2005). Hyperplastic serrated intestinal polyps in humans and in our mouse model differ however from nevi in that they are hyper- and not hypoproliferative.

Another surprising finding was the role of *p53* in Braf-dependent intestinal tumorigenesis. *p53* mutations were reported to be relatively rare in human serrated cancers. Although these studies were based on low sample numbers, many authors assumed that *p53* alterations do not play a role in the “serrated route” of intestinal tumorigenesis (Jass, 2007), thereby contrasting classic colorectal cancer development. Our data clearly demonstrate that this assumption has to be revisited. We show that *p53* mutations accumulate spontaneously in Braf^V637E^-induced murine tumors and provide functional in vivo evidence for its tumor suppressive function. These results are supported by a recent large study, describing that nearly 30% of the 141 examined *BRAF* mutant human CRCs have *p53* mutations (Bond et al., 2011).

Two main findings established the late-stage specificity of p53 tumor suppression. First, selective pressure for *p53* inactivation developed during advanced but not early tumorigenesis. Second, experimental p53 inactivation in *Vil-Cre;Braf*^*LSL-V637E*/+^ mice promoted invasion and metastasis but did not affect adenoma initiation. Mechanistically, our studies suggest a model in which low-level oncogenic signaling observed during early stages of tumorigenesis can drive proliferation, but is insufficient to substantially induce *p19*^*Arf*^ or *p16*^*Ink4a*^. It is only during dysplasia progression when oncogenic signaling exceeds critical thresholds that these tumor suppressors are substantially activated. Dose-dependent effects of oncogenes have been recently observed in breast and lung cancer (Feldser et al., 2010; Junttila et al., 2010; Sarkisian et al., 2007; Murphy et al., 2008), but are in many aspects highly context-dependent. Mapk signaling amplification in Kras^G12D^-driven lung cancer for example occurs at later stages than in our intestinal cancer model (Feldser et al., 2010; Junttila et al., 2010). Dosage effects of oncogene activation probably reflect a mechanism to distinguish physiologic/regenerative from oncogenic growth factor receptor signaling. This discrimination might be of particular importance in the intestine, a highly proliferative organ that is constantly exposed to infectious/toxic damage.

*BRAF* mutations affect a large variety of cancers; however, response rates to Braf inhibitors differ significantly, ranging from 80% in melanoma to less than 10% in *BRAF* mutant CRC (Prahallad et al., 2012). The results of our systematic drug screens in *BRAF* mutant CRC have several implications. First, we demonstrate comparable responses of murine and human *BRAF* mutant tumors to targeted therapeutics, supporting the usefulness of our models for preclinical research. Second, we identified and validated compounds in vivo that overcome resistance to Braf-inhibitor therapy in selected cell lines or across the whole panel, e.g., Mek and combinatorial Braf/PI3K inhibition. Various Mek, PI3K, and BRAF-inhibitors are in late-stage clinical development (Rusconi et al., 2012; Chappell et al., 2011; Flaherty et al., 2012a, 2012b), and our results provide a rationale for their clinical evaluation in *BRAF* mutant CRCs. Third, individual cell lines showed sensitivity to multiple drugs, which often had different targets, suggesting therapeutic options for second- or third-line treatment in tumors that developed resistance to initial regimes. All together these studies show the power of combining genomic information with systematic high-throughput pharmacologic profiling to guide rational therapeutic strategies for specific cancer subentities.

## Experimental Procedures

### Generation of a Conditional Braf^V637E^ Allele

The mouse Braf p.V637E missense mutant protein is the murine counterpart of the human BRAF p.V600E oncogenic variant. V637 is encoded in *Braf* exon 18 (CCDS39463.1), the murine ortholog to human *BRAF* exon 15. Details of allele construction and genotyping protocols are described in the Supplemental Experimental Procedures. Animal protocols were approved by the Home Office (UK) and specified in the Home Office Project License.

### Quantitative PCR

qRT-PCR was performed as described earlier (Rad et al., 2006). Primer/probe sequences are available upon request.

### Methylation Analysis

Methylation-specific PCR was performed upon treatment of DNA with sodium bisulphite. Pyrosequencing reactions and methylation quantification were performed in a PyroMark Q24 System version 2.0.6 (QIAGEN). For detailed description, see the Supplemental Experimental Procedures.

### Sequencing

The coding exons from target genes were enriched either by PCR or via pull down using Agilent SureSelect Mouse Exon Kit. Sequencing was performed using next-generation technologies: Roche 454 GS-FLX (for PCR-amplified *p53* exons) or Illumina HiSeq2000 (for whole-exome sequencing). Calling algorithms will be published elsewhere and are only briefly summarized in the Supplemental Experimental Procedures.

### Histochemistry, Immunohistochemistry, TUNEL Assay, and Western Blotting

Standard techniques were used and are described together with information about antibodies in the Supplemental Experimental Procedures.

### Comparative Genomic Hybridization

CGH array was executed using Agilent 244K mouse whole genome arrays as described previously (Rad et al., 2010).

### MSI Analysis

Microsatellite instability (MSI) was examined using eight microsatellite repeat markers. For details of the method, references, and classification see the Supplemental Experimental Procedures.

### Drug Sensitivity Assays

Drug sensitivity assays were described earlier (Garnett et al., 2012). Specific details used in this study are described in the Supplemental Experimental Procedures.

### Tumor Implantation and Treatment of Mice

Animals were implanted subcutaneously or orthotopically with 1 × 10^7^ cancer cells suspended in culture medium including 50% Matrigel (Beckton Dickinson). Treatment was started when tumors were palpable. Animals were treated once daily with vehicle or 25 mg/kg/day of PD0325901 or a combination of PLX4720 50 mg/kg/day once daily plus GDC09041 75mg/kg/day twice daily by oral gavage for up to 15 days, as indicated in the figure legends. Treatment of endogenous tumors in *Vil-Cre;Braf*^*LSL-V637E/+*^ mice was performed for 5 days.

## Accession Numbers

The EBI European Nucleotide Archive accession number for data presented in this study is PRJEB3418.
