# Supplementary material for: A Genetic Progression Model of BrafV600E-Induced Intestinal Tumorigenesis Reveals Targets for Therapeutic Intervention
Source: Cancer Cell. 2013 Jul 8;24(1):15–29. doi: 10.1016/j.ccr.2013.05.014 (PMC3706745; doi:10.1016/j.ccr.2013.05.014)
Supplement: Document S1. Supplemental Experimental Information, Figures S1–S6, and Tables S1–S7 [file mmc1.pdf]

Cancer Cell, Volume *24*

Supplemental Information

A Genetic Progression Model of Braf^{V600E}-Induced Intestinal Tumorigenesis Reveals Targets for Therapeutic Intervention

Roland Rad, Juan Cadiñanos, Lena Rad, Ignacio Varela, Alexander Strong, Lydia Kriegl, Fernando Constantino-Casas, Stefan Eser, Maren Hieber, Barbara Seidler, Stacey Price, Mario F. Fraga, Vincenzo Calvanese, Gary Hoffman, Hannes Ponstingl, Günter Schneider, Kosuke Yusa, Carolyn Grove, Roland M. Schmid, Wei Wang, George Vassiliou, Thomas Kirchner, Ultan McDermott, Pentao Liu, Dieter Saur, and Allan Bradley

Inventory of Supplemental Information

Supplemental Data

Figure S1, related to Figure 1
Figure S2, related to Figure 2
Table S1, related to Figure 2
Table S3, related to Figure 3
Table S4, related to Figure 3
Figure S3, related to Figure 4
Table S4, related to Figure 4
Table S5, related to Figure 4
Figure S4, related to Figure 5
Figure S5, related to Figure 6
Table S6, related to Figure 6
Figure S6, related to Figure 7
Table S7, related to Figure 7

Supplemental Experimental Procedures

Supplemental References

Supplementary data

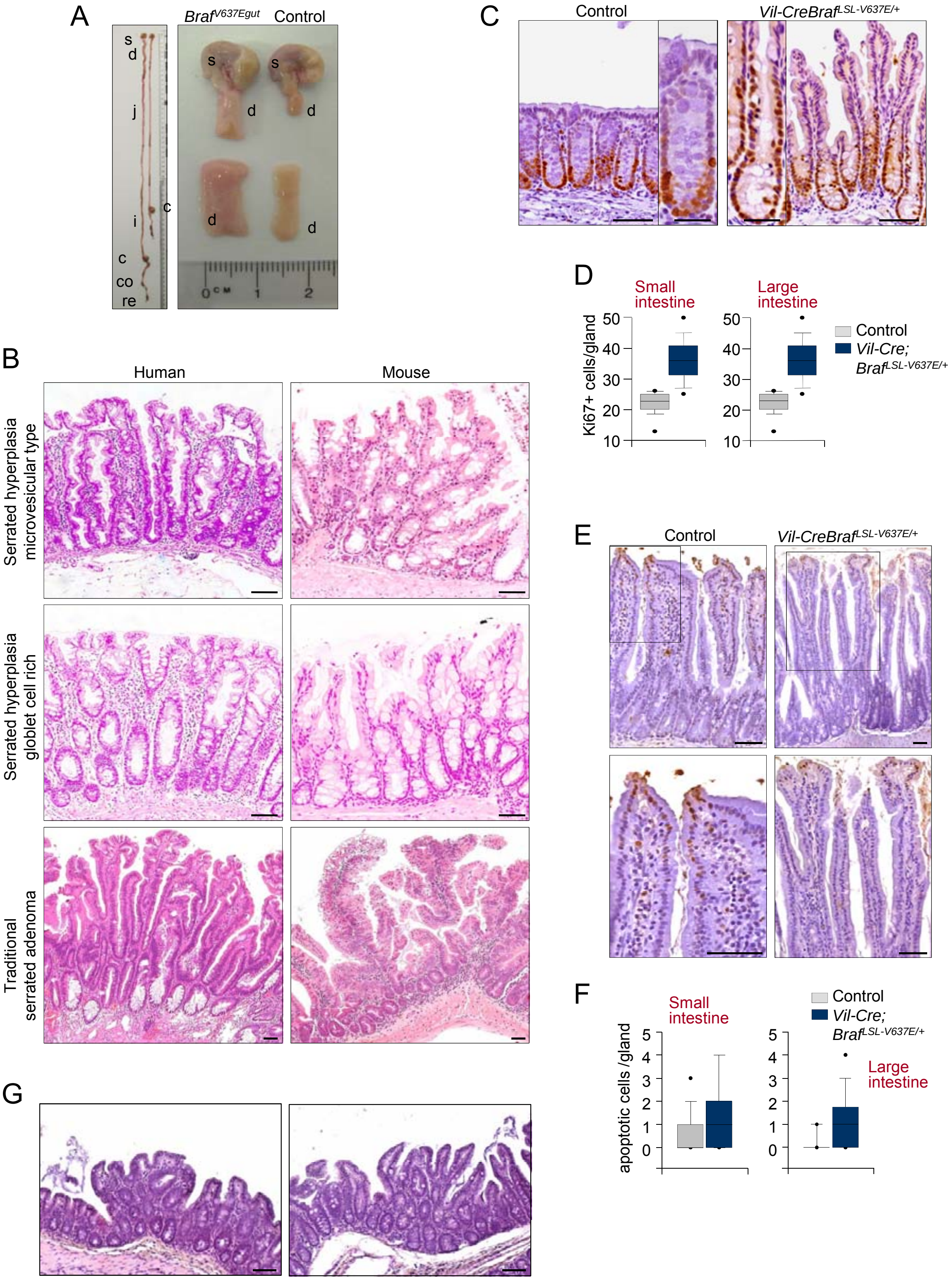

Figure S1, related to Figure 1. Intestinal hyperplasia development in *Vil-Cre;Braf*$^{LSL\text{-}V637E/+}$ mice.

(A) Elongation and thickening of intestines in *Vil-Cre;Braf*$^{LSL\text{-}V637E/+}$ mice. Left image: Representative intestines from a *Vil-Cre;Braf*$^{LSL\text{-}V637E/+}$ mouse (left) and a wild-type control mouse (right). Right image: Stomach and duodenum from a *Vil-Cre;Braf*$^{LSL\text{-}V637E/+}$ mouse (left) and a *Braf* wild-type control mouse (right). s, stomach; d, duodenum; j, jejunum; i, ileum; c, coecum; co, colon; r, rectum;

(B) Human serrated polyps and equivalent murine pathology in *Vil-Cre;Braf*$^{LSL\text{-}V637E/+}$ mice. Murine serrated hyperplasia has high morphologic analogy to *BRAF*-mutant serrated polyps in humans and can be typed as microvesicular (top panels) or goblet-cell rich (middle panels). Murine dysplasia (bottom right panel) resembles traditional serrated adenoma in humans (bottom left) with tubulo-villous architecture and atypical serrated epithelium. Scale bars: 50 µm.

(C-D) Increased proliferation in hyperplastic intestines of *Vil-Cre;Braf*$^{LSL\text{-}V637E/+}$ mice. (C) Ki67 IHC shows that positive cells are restricted to the lower crypt in large intestines of wild-type control mice (left image). In large intestines from *Vil-Cre;Braf*$^{LSL\text{-}V637E/+}$ mice (right image) the proliferative zone is expanded to the mid and upper parts of the crypts. (D) Graphs show the number of Ki67-positive cells per gland in the small and large intestines of mice with indicated genotypes. n>45 glands in ≥ 4 mice per group. There was a significant difference between groups, in both, the small and the large intestine ($p<0.001$; Mann-Whitney Rank Sum Test). Scale bars: 50 µm, 20 µm for insets.

(E-F) Apoptosis is not reduced in mice expressing Braf^{V637E} in the intestine. (E) Apoptotic cells were enumerated after TUNNEL staining in small intestines of *Braf*$^{LSL\text{-}V637E}$ control and *Vil-Cre;Braf*$^{LSL\text{-}V637E/+}$ mice. (F) Plots represent data from three to six mice per group. Thirty crypts/villli were evaluated per mouse. There was no significant difference between groups (Mann-Whitney Rank Sum test). Scale bars: 50 µm.

(G) Sporadic activation of Braf^{V637E} induces focal hyperplastic poylps. *Lgr5-EGFP-IRES-CreERT2* knock-in mice were used to induce stochastic activation of the Braf^{V637E} allele in stem cells*. Lgr5-EGFP-IRES-CreERT2;Braf*$^{LSL\text{-}V637E}$ animals develop hyperplastic polyps as opposed to diffuse hyperplasia in *Vil-Cre;Braf*$^{LSL\text{-}V637E/+}$ mice. Two examples of H&E stained hyperplastic polyps of the large intestine are shown. Scale bars: 50 µm.

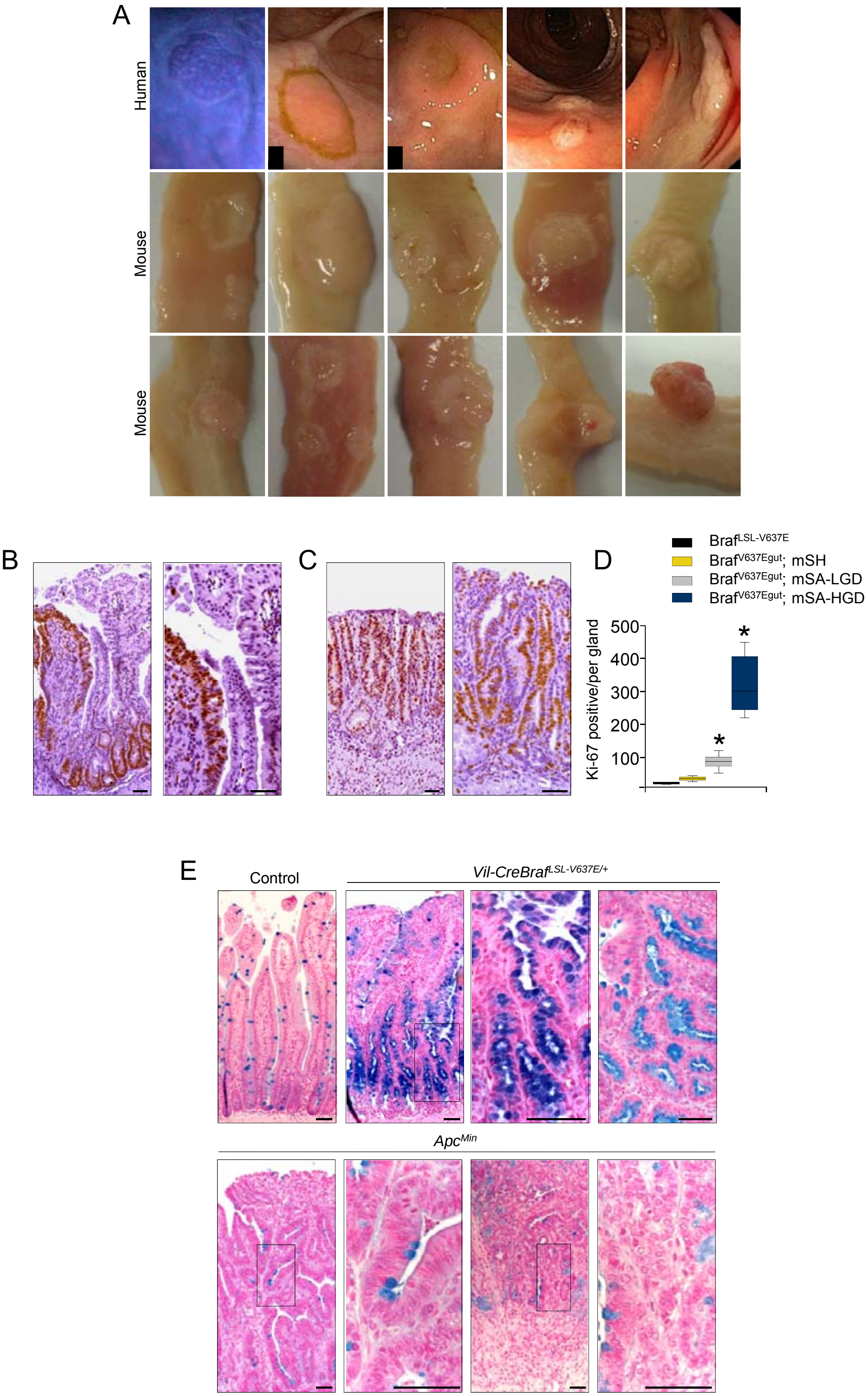
A
Human
Mouse
Mouse
B
C
D
Braf^LSL-V637E
Braf^V637Egut; mSH
Braf^V637Egut; mSA-LGD
Braf^V637Egut; mSA-HGD
Ki-67 positive/per gland
500
400
300
200
100
*
*
E
Control
Vil-CreBraf^LSL-V637E/+
Apc^Min

Figure S2, related to Figure 2. Adenoma and carcinoma development in *Vil-Cre;Braf*$^{LSL\text{-}V637E/+}$ mice.

(A) Macroscopic appearance of Braf-induced tumors. Tumors in *Vil-Cre;Braf*$^{LSL\text{-}V637E/+}$ mice are flat adenomas or carcinomas (Ca) and resemble human sessile tumors. A polypoid mouse tumor is shown as a comparison in the bottom right image. In the top left image the colon mucosa was stained with methylene blue during endoscopy.

(B-D) Increased proliferation in Braf-induced murine dysplasia. (B) Ki-67 staining of a murine serrated adenoma next to a hyperplastic villus from a *Vil-Cre;Braf*$^{LSL\text{-}V637E/+}$ mouse. (C) Positive Ki-67 staining extending from the base of the crypt to the apex of the villi in two mSAs with low-grade adenomas. Some areas have only minor cytological changes but stain posititve for Ki67. All images were taken in small intestines. Scale bars: 50 μm. (D) Number of Ki-67-positive cells per gland in small intestines from indicated mouse genotypes and histological entities. Cells were enumerated in a total of 135 areas in small intestines from 3-5 mice per group. mSH, murine serrated hyperplasia; mSA-LGD, murine serrated adenoma with low-grade dysplasia; mSA-HGD, murine serrated adenoma with high-grade dysplasia. *p<0.001, Mann-Withney Rank Sum test, compared to mSH in *Vil-Cre;Braf*$^{LSL\text{-}V637E/+}$ mice.

(E) *Braf*^{V637E}–induced tumors have a mucinous phenotype. Alcian-blue was used for mucin staining in wild-type small intestinal tissue, *Braf*^{V637E}–induced small intestinal tumors or *Apc*-mutant tumors as indicated. Scale bars: 50 μm.

Table S1, related to Figure 2. Summary of major tumor phenotypes in different cohorts of mice.

Gentype	Phenotype	Description
Vil-Cre; $Braf^{LSL\text{-}V637E/+}$	Hyperplasia	Generalized, serrated, proximal to distal gradient in both the small and the large intestine
	Dysplasia	Murine serrated adenomas (mSA), resembling Tranditional serrated adenomas in humans.<br>Macroscopic mSAs in 96,6% (28/29) of mice aged above 10 months<br>Average number of macroscopic mSAs in mice aged above 10 months: 2.93
	Carcinoma	In one out of 12 mice younger than 10 months<br>In 13.8% (4/29) mice aged above 10 months
	Metastasis	In one out of five mice with cancer, to mesenteric Lymph nodes
Vil-Cre; $Braf^{LSL\text{-}V637E/+}$; $p53^{LSL\text{-}R172H/+}$	Hyperplasia	Generalized, serrated, proximal to distal gradient in both the small and the large intestine
	Dysplasia	Murine serrated adenomas (mSA), resembling Tranditional serrated adenomas in humans.<br>Marcoscopic mSAs in 100% (18/18) of mice aged above 10 months<br>Average number of macroscopic mSAs in mice aged above 10 months: 2.38
	Carcinoma	In 2 out of 11 mice (18.2%) younger than 10 months<br>In 55.6% (10/18) mice aged above 10 months; two mice had multiple synchronous cancers
	Metastasis	In 3 out of 12 mice with cancer, to (1) lung, (2) pancreas and mesenteric lymph nodes and (3) liver.
Vil-Cre; $Braf^{LSL\text{-}V637E/+}$; $p16^{*}$	Hyperplasia	Generalized, serrated, proximal to distal gradient in both the small and the large intestine
	Dysplasia	Murine serrated adenomas (mSA), resembling Tranditional serrated adenomas in humans.<br>Marcoscopic mSAs in 97.1% (33/34) of mice aged above 10 months<br>Average number of macroscopic mSAs in mice aged above 10 months: 4.24
	Carcinoma	In 3 out of 22 mice (13.6%) younger than 10 months<br>In 58.8% (20/34) mice aged above 10 months; 10 mice had multiple synchronous cancers
	Metastasis	In 3 out of 12 mice with cancer, to local lymph nodes (Tumors 1-3) and stomach, liver, lungs (Tumor 1)

*p16 heterozygous or homozygous mutants were pooled

Table S2, related to Figure 3. Comparison of mSA (murine serrated adenoma with dysplasia) development in *Vil-Cre;Braf*$^{LSL-V637E/+}$ and *Vil- Cre;Braf*$^{LSL-V637E/+}$*;p53*$^{LSL-R172H/+}$ mice.

Genotype	Age group	No of mice	No of tumors	Mean	p value[#]	No of mice with tumors	Percent mice with tumors	p value[*]
Vil-Cre;Braf$^{LSL-V637E/+}$	All	41	95	2.32	0.23	34	82.9	0.99
Vil- Cre;Braf$^{LSL-V637E/+}$*;p53*$^{LSL-R172H/+}$		29	51	1.76		24	82.8	
Vil-Cre;Braf$^{LSL-V637E/+}$	<300 d	12	10	0.83	0.92	6	50.0	0.83
Vil- Cre;Braf$^{LSL-V637E/+}$*;p53*$^{LSL-R172H/+}$		11	8	0.73		6	54.5	
Vil-Cre;Braf$^{LSL-V637E/+}$	>300 d	29	85	2.93	0.53	28	96.6	0.43
Vil- Cre;Braf$^{LSL-V637E/+}$*;p53*$^{LSL-R172H/+}$		18	43	2.39		18	100.0	

Light grey: comparison of the total number of tumors between groups. Dark grey: comparison of the number of mice with tumors between groups. Only macroscopic tumors (larger than 2 mm) were included in the analysis. For these statistical comparisons of mSA development in different gentotype groups, the number of mSAs was defined as the total number of tumors. This accounts for the fact that carcinomas develop trough a dysplastic stage. [#]p values were calculated by a Mann-Whitney Rank Sum test. [*]p values were calculated by a χ^2 test.

Table S3, related to Figure 3. Comparison of carcinoma development in *Vil-Cre;Braf*$^{LSL-V637E/+}$ and *Vil-Cre;Braf*$^{LSL-V637E/+}$*;p53*$^{LSL-R172H/+}$ mice.

Genotype	Age group	No of mice	No of tumors	Mean	p value[#]	No of mice with Tus	Percentage of mice with tumors	p value[*]
Vil-Cre;Braf$^{LSL-V637E/+}$	All	41	5	0.122	0.004	5	82.9	0.005
Vil- Cre;Braf$^{LSL-V637E/+}$*;p53*$^{LSL-R172H/+}$		29	15	0.517		12	41.4	
Vil-Cre;Braf$^{LSL-V637E/+}$	<300 d	12	1	0.08	0.37	1	8.3	0.48
Vil- Cre;Braf$^{LSL-V637E/+}$*;p53*$^{LSL-R172H/+}$		11	2	0.18		2	18.2	
Vil-Cre;Braf$^{LSL-V637E/+}$	>300 d	29	4	0.14	0.007	4	13.8	0.002
Vil- Cre;Braf$^{LSL-V637E/+}$*;p53*$^{LSL-R172H/+}$		18	13	0.722		10	55.6	

Light grey: comparison of the total number of cancers between groups. Dark grey: comparison of the number of mice with cancers between groups. In blue is a comparision of the number of mice with cancers between groups. [#]p values were calculated by a Mann-Whitney Rank Sum test. [*]p values were calculated by a χ^2 test.

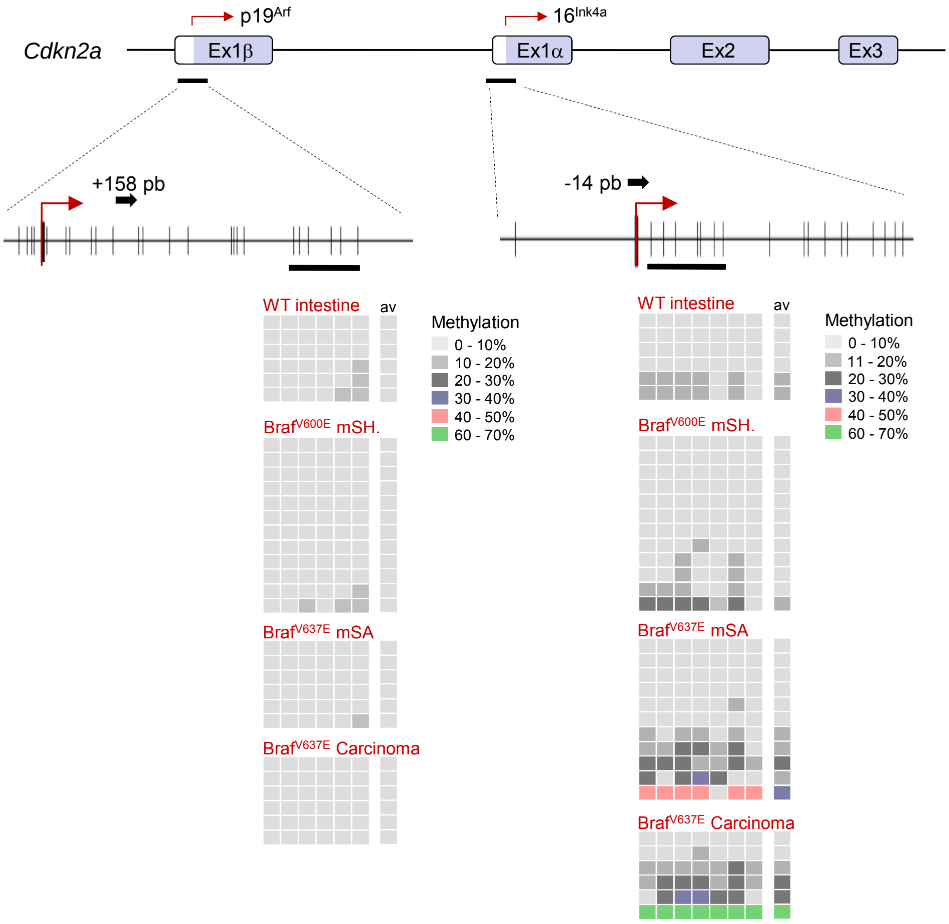

Figure S3, related to Figure 4. CpG methylation analysis in the *p19*^{Arf} and *p16*^{Ink4a} promoter regions in indicated sample sets. The level of methylation was analyzed at indicated CpGs using pyrosequencing. Each column represents one CpG. Rows represent individual samples. mSH, murine serrated hyperplasia; mSA, murine serrated adenoma with dysplasia.

Table S4, related to Figure 4. Comparison of mSA (murine serrated adenoma with dysplasia) development in *Vil-Cre;Braf*$^{LSL\text{-}V637E/+}$ and *Vil-Cre;Braf*$^{LSL\text{-}V637E/+}$*;p16** mice.

Genotype	Age group	No of mice	No of tumors	Mean	p value#	No of mice with tumors	Percent mice with tumors	p value£
Vil-Cre;Braf$^{LSL\text{-}V637E/+}$	All	41	95	2.317	0.33	34	82.9	0.59
Vil-Cre;Braf$^{LSL\text{-}V637E/+}$*;p16**		56	171	3.054		44	78.6	
Vil-Cre;Braf$^{LSL\text{-}V637E/+}$	<300 d	12	10	0.83	0.62	6	50.0	1
Vil-Cre;Braf$^{LSL\text{-}V637E/+}$*;p16**		22	27	1.23		11	50.0	
Vil-Cre;Braf$^{LSL\text{-}V637E/+}$	>300 d	29	85	2.93	0.08	28	96.6	0.91
Vil-Cre;Braf$^{LSL\text{-}V637E/+}$*;p16**		34	144	4.24		33	97.1	

*P16** indicates all *p16*-mutant mice (homozygous and heterozygous). Light grey: comparison of the total number of tumors between groups. Dark grey: comparison of the number of mice with tumors between groups. Only macroscopic tumors (larger than 2 mm) were included in the analysis. For these statistical comparisons of mSA development in different genotype groups, the number of mSAs was defined as the total number of tumors to account for the fact that carcinomas develop trough a dysplastic stage. #p values were calculated by Mann-Whitney Ranks Sum test. £p values were calculated by a χ^2 test.

Table S5, related to Figure 4. Comparison of carcinoma development in *Vil-Cre;Braf*$^{LSL\text{-}V637E/+}$ and *Vil-Cre;Braf*$^{LSL\text{-}V637E/+}$*;p16** mice. P16* indicates all p16-mutant mice (homozygous and heterozygous). The upper panel compares the total number of cancers between groups. The lower panel compares the number of mice with cancers between groups.

Genotype	Age group	No of mice	No of tumors	Mean	p value#	No of mice with tumors	Percent mice with tumors	p value£
Vil-Cre;Braf$^{LSL\text{-}V637E/+}$	All	41	5	0.12	<0.001	5	12.2	0.002
Vil-Cre;Braf$^{LSL\text{-}V637E/+}$*;p16**		56	44	0.79		23	41.1	
Vil-Cre;Braf$^{LSL\text{-}V637E/+}$	<300 d	12	1	0.08	0.68	1	8.3	0.65
Vil-Cre;Braf$^{LSL\text{-}V637E/+}$*;p16**		22	3	0.14		3	13.6	
Vil-Cre;Braf$^{LSL\text{-}V637E/+}$	>300 d	29	4	0.14	<0.001	4	13.8	0.0002
Vil-Cre;Braf$^{LSL\text{-}V637E/+}$*;p16**		34	41	1.21		20	58.8	

P16* indicates all *p16*-mutant mice (homozygous and heterozygous). Light grey: comparision of the total number of cancers between groups. Dark grey: comparison of the number of mice with cancers between groups. #p values were calculated by Mann-Whitney Ranks Sum test. £p values were calculated by a χ^2 test.

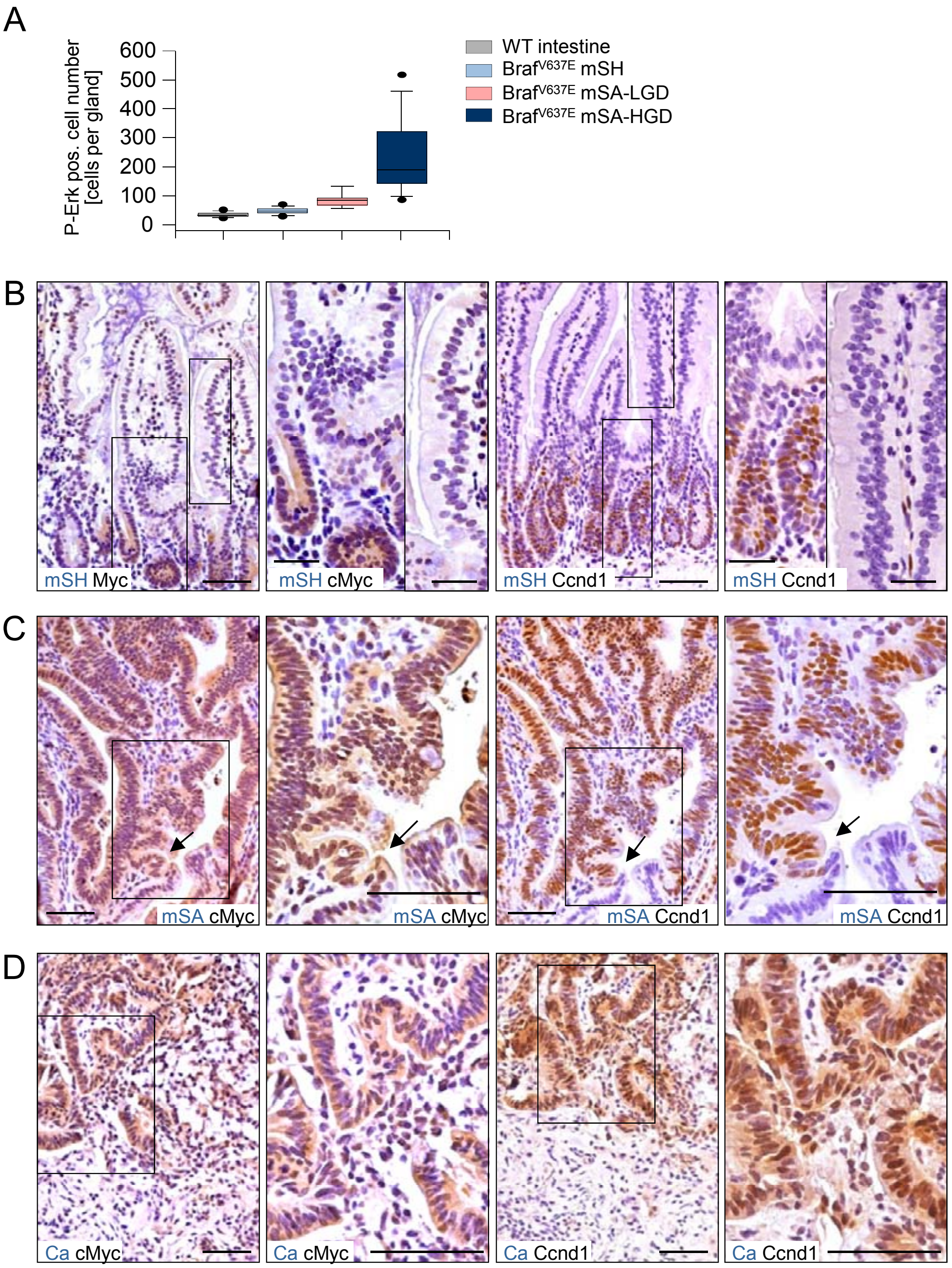

Figure S4, related to Figure 5. Map kinase pathway activation during Braf-induced tumor progression.

(A) Quantification of pERK-positive cells in tissue samples with the indicated histology and genotypes. Phospho-Erk-positive cells were enumerated following IHC staining in a total of 168 areas in small intestines from 5-8 mice per group. $p<0.001$ for all groups as compared to wild-type intestine; Mann-Whitney Rank Sum test. mSH, murine serrated hyperplasia; mSA-LGD, murine serrated adenoma with low-grade dysplasia; mSA-HGD, murine serrated adenoma with high-grade dysplasia.

(B-D) Immunoreactivity for cMyc and Cyclin D1 (Ccnd1) at different stages of Braf-induced tumorigenesis. IHC staining for cMyc and Ccnd1 is shown in murine $Braf^{V637E}$-induced hyperplasia (B), dysplasia (C) or carcinoma (D). In hyperplasia cMyc and Ccnd1 expression is restricted to the proliferative zone. Note the different cMyc and Ccnd1 immunoreactivity in high-grade dysplasia and hyperplasia. Transition zones are indicated with arrows in (C). mSH, murine serrated hyperplasia; mSA, murine serrated adenoma with dysplasia; Ca, carcinoma. Scale bars: 50 μm.

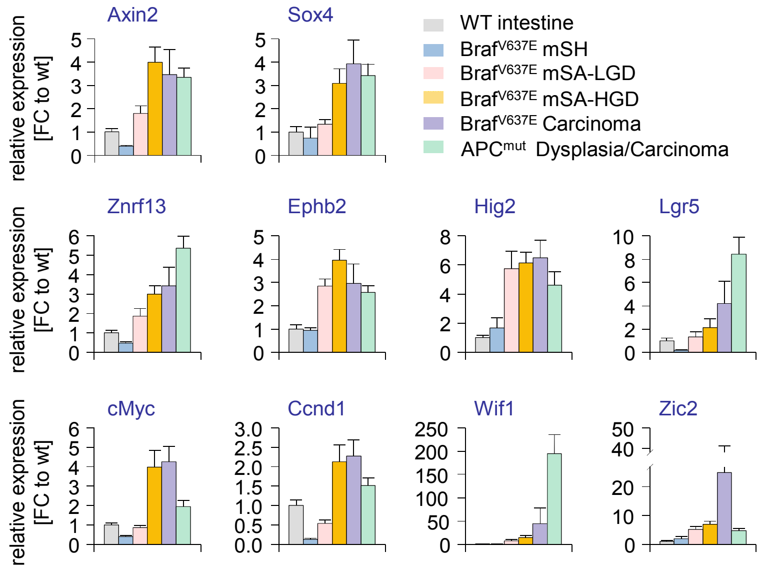

Figure S5, related to Figure 6. Expression of Wnt target genes during Braf-induced tumorigenesis. Wnt target gene expression is shown for small intestinal samples with the indicated histology and genotypes. Relative mRNA expression was determined by qRT-PCR and normalized to Gapdh expression. Data are shown as fold-change (FC) values as compared to expression in wild-type intestine. A total of 11 wild-type and 53 *Braf*-mutant as well as 14 *Apc*-mutant tissue samples were analyzed. Red lines indicate mean values within groups. Error bars, SEM. mSH, murine serrated hyperplasia; mSA-LGD, murine serrated adenoma with low-grade dysplasia; mSA-HGD, murine serrated adenoma with high-grade dysplasia.

Table S6, related to Figure 6. Mutations in Wnt pathway genes in Braf^{V637E}-induced murine intestinal tumors.

Sample	Patho-logy	Chr	Position	Ref Base	Mut Base	Gene	c.Annot	p.Annot	Effect	Type	Geno-type	Gene function
4.6aT2	HGD	5	135726884	G	T	*Fzd9*	c.16 C>A	p.L6M	Missense	Subst	VBP53	Wnt receptor
12.2cT4	CA	2	40860206	A	G	*Lrp1b*	c.6964 T>C	p.S2322P	Missense	Subst	VBP16	Wnt receptor
2.5xT1	HGD	2	40557325	G	A	*Lrp1b*	c.11575 C>T	p.R3859C	Missense	Subst	VBP53	Wnt receptor
4.1cT1	HGD	2	41049175	G	C	*Lrp1b*	c.5476 C>G	p.Q1826E	Missense	Subst	VB	Wnt receptor
4.2fT2	HGD	2	41101487	T	A	*Lrp1b*	c.5425 A>T	p.T1809S	Missense	Subst	VBP53	Wnt receptor
4.6aT1	HGD	2	40714015	A	C	*Lrp1b*	c.9425 T>G	p.I3142S	Missense	Subst	VBP53	Wnt receptor
5.2eT1	CA	2	40555148	A	C	*Lrp1b*	c.11627 T>G	p.L3876R	Missense	Subst	VBP53	Wnt receptor
12.2cT4	CA	2	91326073	G	A	*Lrp4*	c.2378 G>A	p.S793N	Missense	Subst	VBP16	Wnt receptor, antagonistic
8.1aT4	CA	4	107523942	-	G	*Lrp8*	c.757 ->G	p.G253fs*8	Frameshift	INDEL	VB	Wnt receptor
4.2fT1	HGD	2	109836895	G	A	*Lgr4*	c.586 G>A	p.A196T	Missense	Subst	VBP53	Wnt receptor
4.1dT2	HGD	18	34473817	-	C	*Apc*	c.4111 ->C	p.P1371fs*23	Frameshift	INDEL	VB	intracellular Wnt pathway component
4.2aT2	HGD	18	34473808	-	C	*Apc*	c.4102 ->C	p.P1368fs*26	Frameshift	INDEL	VB	intracellular Wnt pathway component
5.2bT3	HGD	18	34432092	G	T	*Apc*	Exon 5 - 1 G>T	0	Ess. splice site	Subst	VB	intracellular Wnt pathway component
5.3cT2	HGD	18	34473817	-	C	*Apc*	c.4111 ->C	p.P1371fs*23	Frameshift	INDEL	VBP53	intracellular Wnt pathway component
5.6bT1	HGD	18	34472266	C	T	*Apc*	c.2560 C>T	p.R854*	Nonsense	Subst	VBP53	intracellular Wnt pathway component
8.1aT4	CA	18	34473712	C	T	*Apc*	c.4006 C>T	p.Q1336*	Nonsense	Subst	VB	intracellular Wnt pathway component
12.2cT4	CA	9	120859748	C	T	*Ctnnb1*	c.122 C>T	p.T41I	Missense	Subst	VBP16	intracellular Wnt pathway component
12.2cT7	CA	9	120859723	T	G	*Ctnnb1*	c.97 T>G	p.S33A	Missense	Subst	VBP16	intracellular Wnt pathway component
3.2dT1	CA	9	120859748	C	T	*Ctnnb1*	c.122 C>T	p.T41I	Missense	Subst	VBP16	intracellular Wnt pathway component
3.6cT3	HGD	9	120859726	G	A	*Ctnnb1*	c.100 G>A	p.G34R	Missense	Subst	VBP16	intracellular Wnt pathway component
3.6cT4	CA	9	120859748	C	T	*Ctnnb1*	c.122 C>T	p.T41I	Missense	Subst	VBP16	intracellular Wnt pathway component
9.5fT5	HGD	9	120859748	C	T	*Ctnnb1*	c.122 C>T	p.T41I	Missense	Subst	VBP16	intracellular Wnt pathway component
9.6gT1	CA	9	120859748	C	T	*Ctnnb1*	c.122 C>T	p.T41I	Missense	Subst	VBP16	intracellular Wnt pathway component
2.5xT1	HGD	9	120859736	C	T	*Ctnnb1*	c.110 C>T	p.S37F	Missense	Subst	VBP53	intracellular Wnt pathway component
3.3iT2	HGD	9	120859724	C	T	*Ctnnb1*	c.98 C>T	p.S33F	Missense	Subst	VB	intracellular Wnt pathway component
9.5fT1	CA	16	38228779	C	T	*Gsk3b*	c.1112 C>T	p.P371L	Missense	Subst	VBP16	intracellular Wnt pathway component
4.6aT2	HGD	11	108803846	C	A	*Axin2*	c.1542 C>A	p.H514Q	Missense	Subst	VBP53	intracellular Wnt pathway component

Data were obtained as follows: Whole-exome sequencing was performed in 20 tumors and the most frequently mutated Wnt pathway genes *Lrp1b*, *Apc* and *Ctnnb1* were analyzed in an additional set of 46 tumors.*LGD, low grade dysplasia; HGD, high-grade dysplasia, CA, carcinoma. VB, *Vil-Cre;Braf*$^{LSL\text{-}V637E/+}$; VBP53, *Vil-Cre;Braf*$^{LSL\text{-}V637E/+}$;*p53*$^{LSL\text{-}R172H+/-}$; VBP16, *Vil-Cre;Braf*$^{LSL\text{-}V637E/+}$;*p16*;*

A Non-treated (control) B PD0325901-treated

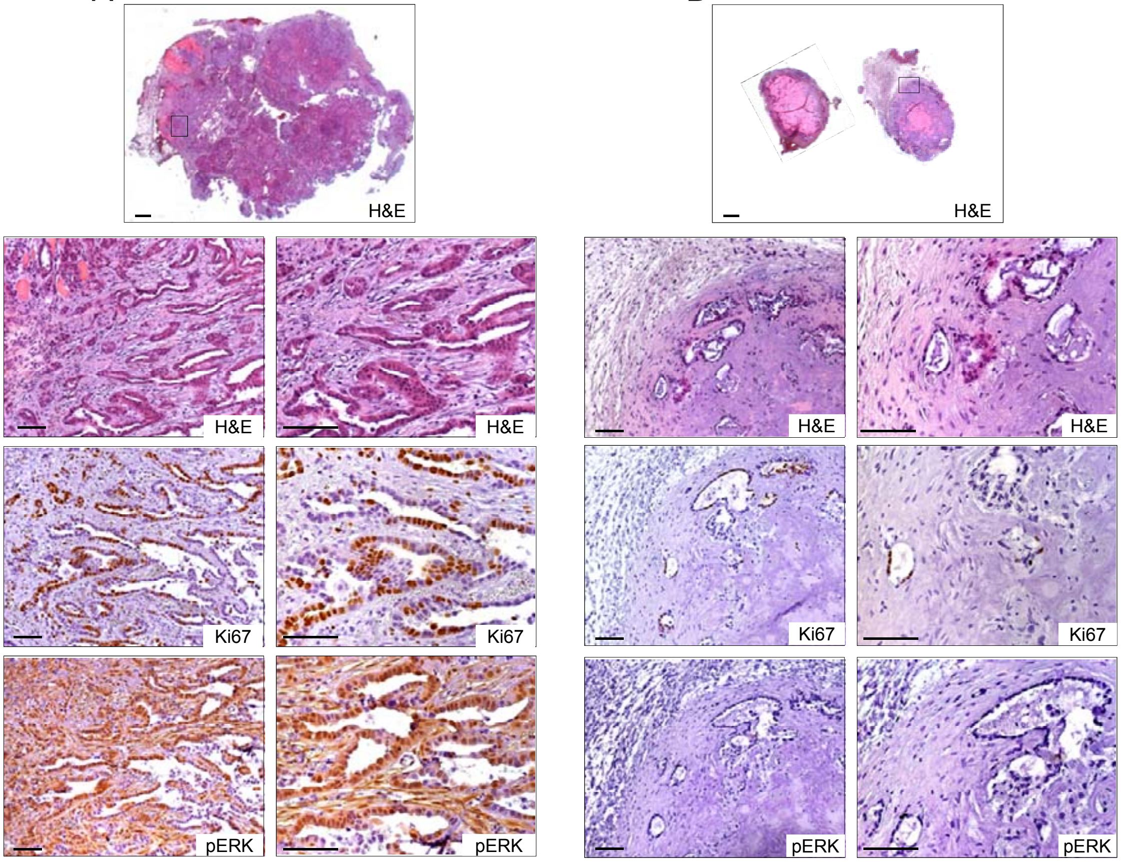

C

Mouse ID	Treamtent group	Tumour size (cm^3)	Tumour weight (g)	Other observations
1	control	1.00	0.61	Haemorrhagic ascites LN metastases
2	control	0.32	0.46	-
3	control	0.11	0.04	LN metastases
4	PD0325901	0.01	0.01	-
5	PD0325901	no tumor visible	no tumor visible	-
6	PD0325901	no tumor visible	no tumor visible	-

D Non-treated (control) PD0325901-treated

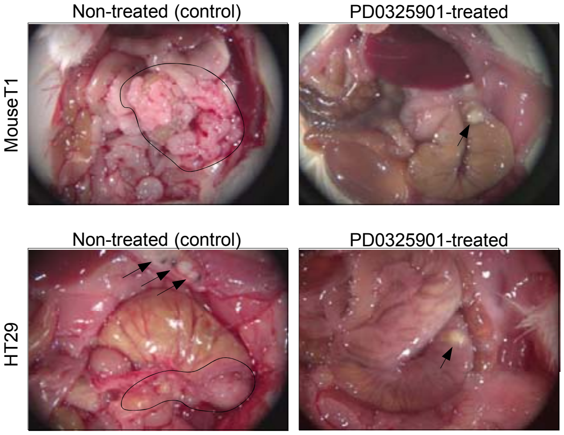

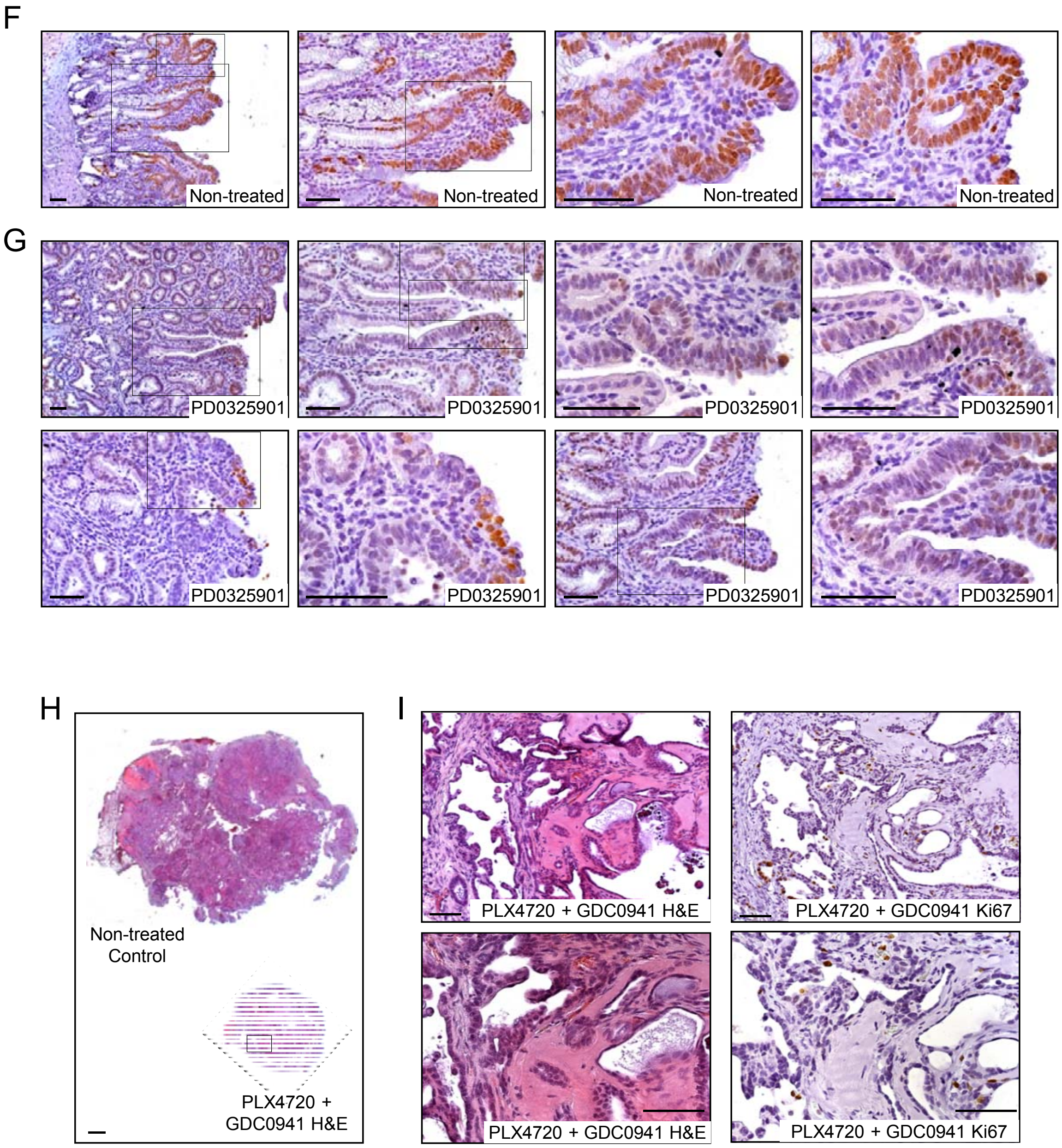

Figure S6, related to Figure 7. Pharmacologic inhibition of tumor growth in vivo.

(A-B) Treatment of allo- and xenotransplanted *Braf*-mutant intestinal cancer cell lines with the Mek inhibitor PD0325901 inhibits Erk phosphorylation and proliferation and induces near-complete tumor regression. H&E and IHC staining for Ki67 and phospho-Erk of a representative non-treated (A) and 2 treated (B) subcutaneous allografts of a murine cancer cell line derived from $Braf^{V637E}$-mutant intestinal cancer (MouseT1). Cell were transplanted into NSG (Nod Scid $IL12Rg^{null}$) mice. Treatment was started when tumors were palpable. Animals were treated once daily with vehicle (Control) or 25mg/kg/d of PD0325901 for 15 days by oral gavage. Note the large eosinophilic areas without cancer cells in the treated tumors, showing that although the tumor mass is still detectable macroscopically (size for both tumors 3mm) it is largely composed of connective or necrotic tissue. Scale bars, 500 µm for top micrographs in panels A and B, 50 µm in all other panels.

(C-E) The Mek inhibitor PD0325901 inhibits growth and metastasis of orthothopically transplanted Braf-mutant intestinal cancer cell lines. Orthotopic transplants of *Braf*-mutant MouseT1 (C/D) and human HT29 (E) cell lines into the cecum of NSG (Nod Scid IL12R-gamma^{null}) mice. The table shows the characteristics of tumor growth and metastasis in vehicle treated (Control) and PD0325901-treated mice implanted with MouseT1. Treatment with the Mek-inhibitor PD0325901 was started 14 days after implantation and was performed for 17 days at a dose of 25mg/kg/d. Examples for tumor growth in non-treated and treated mice is shown.

(F/G) PD0325901 inhibits proliferation of endogenous Braf^{V637E}-induced intestinal tumors. *Vil-Cre;Braf$^{V637E/+}$* mice were treated with either vehicle or PD0325901 for 5 days at a dose of 25mg/kg/d. Ki67 staining is shown in dysplasia from vehicle-treated *Vil-Cre;Braf$^{V637E/+}$* control mice (F) and three tumors from PD0325901-treated animals (G). Scale bars: 50 µm, in magnifications 20 µm.

(H/I) Combinatorial PI3K and Braf treatment of transplanted *Braf*-mutant intestinal cancer cell lines inhibits tumor growth. H&E stained examples of non-treated (upper tumor in H) and treated (lower tumor in H and panels in I) subcutaneous allograft transplants of a MouseT1 cell line (derived from a Braf^{V637E}-mutant murine intestinal cancer) into NSG (Nod Scid IL12Rg^{null}) mice. Treatment was started when tumors were palpable. Animals were treated with vehicle (Control) or a combination of PLX4720 50mg/kg/d once daily plus GDC09041 75mg/kg/d twice daily by oral gavage. Note the large eosinophilic areas without cancer cells in the treated tumors. Ki67 staining (G) shows a lack of proliferation in the majority of surviving tumor cells in the PXL4720/GDC0941 treated tumor. For comparative reasons, the same non-treated control tumor is shown here and in (A). Scale bars, 500 µm (H), 50 µm (I).

Table S7, related to Figure S7. Concentrations of compounds used as single agents or in combination with 0.5 μM PLX4720 for in vitro drug sensitivity screening.

Compound	Conc (μM)	Target
PLX4720	0.5	Braf
GSK1904529A	0.2	IGF-1R/IR
Sunitinib	1	PDGFRA, KIT
VX-680 (MK-0457)	0.2	AURK A/B/C, ABL, FLT3
Imatinib	0.2	Abl, KIT, PDGFR
TAE684	0.2	ALK
PF2341066	0.2	MET, ALK
Dasatanib	0.05	Src, Bcr-Abl
XAV 939	1	TNKS1/2 (Wnt/b-catenin)
Gefitinib	0.2	EGFR
ABT-263	0.5	Bcl-2, Bcl-xl
SAHA (Vorinostat)	0.5	HDAC I/II
AZD2281 (Olaparib)	1	PARP1/2
Lenalidomide	1	TNF alpha
Axitinib	0.5	PDGFR, KIT, VEGFR
AZD 7762	0.2	Chk 1/2
Gefitinib	0.5	EGFR
CEP-701 (Lestaurtinib)	0.1	JAK2, FLT3
CHIR-99021	1	GSK-3
17-AAG	0.05	Hsp90
VX-702	1	p38
KU-55933	0.2	ATM
Elesclomol	0.005	Hsp70 inducer
BIBW 2992	0.1	EGFR, ERBB2
GDC-0449	2	SMO (Hedgehog)
BX 795	1	PDK1, TBK1, IKKe
SL 0101-1	2	Rsk, AURKB, PIM3
BIRB 0796	2	p38, JNK2
JNK inhibitor VIII	2	JNK 1/2/3
PD173074	0.2	FGFR
ZM447439	0.5	AURK B
MK-2206	1	AKT
PD-0332991	0.5	CDK 4/6
GW843682X (AN-13)	0.05	Plk1
NVP-BEZ235	0.01	PI3K/mTOR
GDC0941	1	PI3K (a,b,d)
AZD8055	0.5	mTORC1/2
SB590885	0.5	BRAF
AZD6244	0.5	MEK 1/2
WO2009093972	0.2	PI3K beta
AZD6244 + MK-2206	0.5/1	Comb [MEK1/2 + AKT]
GSK 269962A	0.2	ROCK1/2
BMS-708163	0.2	Gamma-secretase
Obatoclax mesylate	0.05	Mcl-1
Nutlin-3	2	MDM2
Embelin	2	XIAP
Rapamycin	0.002	mTOR
BMS-536924	0.5	IGF-1R
SB-505124	1	TGFβR-I (ALK5)
PD0325901	0.005	MEK 1/2

Supplementary Methods.

Mouse strains and animal experimentation. Animal protocols were approved by the Home Office (UK) and specified in the Home Office Project Licence. P53$^{LSL\text{-}R172H/+}$ knock-in mice expressing the mouse equivalent of the dominant-negative human *TP53*^{R175H} mutation conditionally (Olive et al., 2004) and *p16*^{Ink4a*} mice, which have a point mutation that is silent in the *p19*^{Arf} reading frame, but introduces a stop codon in *p16*^{Ink4a} have been described earlier (Krimpenfort et al., 2001).

Generation of conditional *Braf*$^{LSL\text{-}V637E}$ knock-in mice. The mouse Braf p.V637E missense mutant protein is the murine counterpart of the human BRAF p.V600E oncogenic variant, and it is named based on the only consensus coding sequence (CCDS) (Pruitt et al., 2009) currently accepted for mouse Braf: CCDS39463.1. According to CCDS39463.1, V637 is encoded in *Braf* exon 18, the murine orthologue to human *BRAF* exon 15. The details of construction of the allele are available as supplementary information. Genotyping primers are shown below.

Primer Name	Product Size	Sequence
**Genotyping of *Braf*$^{LSL\text{-}V637E/+}$ mice**		
Braf-5	MT: 664bp WT: 399bp	TTTATCATAGTAGGGCTTGCTGTCTTGCTT
Braf-WT3		CAAATATGTTTTGAGCAAGACCTTTGTTCT
En2SAsseq3		CCACTGACCAGAAGGAAAGTGGT
**Detection of LSL recombination at the *Braf* locus**		
Braf_afterCre F	rec: 391bp non-rec: 2kb	CTGATCCACCTGCAGTCAGCTAT
Braf_afterCre R		GGCCCAGGCTCTTTATGAGAATA

Rec, recombined; LSL, lox-stop-lox;

To generate *Braf*$^{LSL\text{-}V637E}$, we first amplified mouse *Braf* exon 18 and its flanking sequences from BAC clone 412C3 using Phusion polymerase (Finnzymes), and cloned it into pBS. The resulting plasmid was used as a template for site directed mutagenesis with the Quikchange multisite directed mutagenesis kit (Stratagene), to introduce the c.1910T>A mutation (orthologous to the human *BRAF* c.1799T>A mutation).

Then, the *Braf*^{V637E} mutation was introduced in the sequence of BAC clone 412C3 through recombineering (Lee et al., 2001; Liu et al., 2003). Subsequently, the genomic region containing the *Braf*^{V637E} mutation flanked by the homology arms was retrieved from the modified 412C3 BAC into pBS-DTA.

In parallel, a series of cloning steps were performed to introduce into pQL2 (a vector originally developed by Qi Liang to place a Sleeping-Beauty repeat into genomic DNA by recombineering) both short homology arms from mouse *Braf* intron 17 (BRSA1 and BRSA2) and a series of elements necessary to insert a removable “trapping” cassette along with the Sleeping Beauty repeat already contained in pQL2. These elements consisted of a Lox2272 site, a splice acceptor from the engrailed-2 mouse locus (En2SA) and 4 polyadenylation sequences from the SV40 virus (4xSV40pA), and they were used to generate pQL2-BRSA1-Lox2272-En2SA-4xSV40pA-BRSA2. This vector was linearized and electroporated into EL-350 recombineering competent E. coli cells (Lee et al., 2001), previously transformed with the pBS-DTA plasmid containing the relevant Braf genomic DNA region with the activating mutation in exon 18. Recombinant constructs were selected by Ampicillin/Kanamycin resistance and retransformed to segregate mixtures of recombinant and non-recombinant plasmids replicating in the same bacterium.

Finally, the BRSA4 short homology arm was PCR amplified using Phusion polymerase and DNA from the 412C3 BAC as template, the BRSA3 short homology arm followed by a Lox2272 site were generated by

ligation of long oligonucleotides, and BRSA4 and BRSA3-Lox2272 were cloned in pZK5-SB-FRT (developed by Qi Liang to introduce another SB repeat into genomic DNA) in a four-way ligation which also contained a pu∆TK cassette extracted from pZK5-SB-FRT.

The resulting plasmid was linearized and electroporated into the EL350 cells generated from the previous step. Kanamycin/chloramphenicol resistant bacteria were selected and the completeness of the resulting targeting construct was checked by digestion with multiple restriction enzymes and capillary sequencing. Large quantities of pure plasmid were obtained by Maxi-prep (Qiagen). NotI linearized targeting vector was electroporated in AB1 ES cells and puromycin resistant clones were selected, expanded and microinjected in blastocysts to generate chimaeric mice, from which *Braf*$^{LSL\text{-}V637E\ /+}$ knock-in progeny were obtained.

Our mutant *Braf* allele is different from conditional *Braf* knock-in alleles published to date in the strategy used to prevent mutant *Braf* expression in the absence of Cre-mediated activation. Both of the two previous models contain a lox flanked minigene created by the fusion of the four last exons of wild-type *Braf* inserted in the intron preceding the exon containing the point mutation (Mercer et al., 2005; Dankort et al., 2007). Non-endogenous splice acceptor and polyadenylation signals flank the minigene. Thus, before activation, wild type *Braf* is expressed from both the wild-type and the knock-in minigene containing alleles. In our case, the intron preceding the mutant *Braf* exon contains a lox-stop-lox cassette preventing its expression. Upon activation, when the floxed minigene and the lox-stop-lox cassettes are removed, both types of alleles should behave very similarly. However, there are differences before activation which deserve mentioning. In the minigene setting, both alleles contribute to normal *Braf* expression, but the transcripts derived from the minigene lack the last three introns and the natural 3'UTR. In our case, before activation, wild-type *Braf* is only expressed from the wild-type allele. While this might cause lower expression of wild-type *Braf* before activation, all wild-type transcripts keep their original structure, and thus are subject to natural post-transcriptional regulation, including potential miRNA or ceRNA mediated transcript level control. Although we do not discard that these features might cause phenotypic differences between our and previous *Braf* mutant knock-in models, we consider these mouse models are most likely equivalent. Finally, one unique feature included into our allele is flanking of the Stop cassette with *Sleeping Beauty* inverted terminal repeats. This allows excision of the cassette by transposition upon expression of the *Sleeping Beauty* transposase, a feature that can be exploited for various applications.

Methylation analysis. Genomic DNA (0.5 µg) isolated from tissues and tumors was modified with sodium bisulphite with the EZ DNA methylation kit (Zymo Research Corporation) following the manufacturer's protocol. Bisulphite-treated DNA was eluted in 15 µl of water and 2 µl were used for each PCR. The set of primers for PCR amplification and sequencing were designed using version 2.0.01.15 of PyroMark assay design software (Qiagen) and are shown below.

Primer Name	Sequence
p16^{Ink4a}-F	AGTAGTGTTTTTAGGGGTGT
p16^{Ink4a}-R	[Btn]-CCATACTACTCCAAATAACTCTC
p16^{Ink4a}-Seq	GGAAGGAGGGATTTATTG
p19^{Arf}-F	AGGTTTTTGGTTATTGTGAGGATTTA
p19^{Arf}-R	[Btn]-CCTCTTCTCAAAATCCTCTCTAAC
p19^{Arf}-Seq	GGTTTTTTTGGTGAAGT

*Btn, biotinylated.

Amplification primers hybridize with CpG-free sites to ensure methylation-independent reaction and one primer (opposite to the sequencing primer) is biotinylated (Btn) to convert the PCR product to single-stranded DNA templates. We used the Vacuum Prep Tool (Biotage, Sweden) to prepare single-stranded PCR products according to the manufacturer's instructions. Pyrosequencing reactions and methylation quantification were performed in a PyroMark Q24 System version 2.0.6 (Qiagen). Graphic representation of methylation values shows color boxes identifying CpG sites that present percentage methylation values as indicated in Figure S3.

Sequencing. Coding exons from target genes were enriched either by PCR *(p53)* or via whole-exome pull down using Agilent SureSelect Mouse Exon Kit according to manufacturer's instructions. Primers for PCR amplification of exons 2 to 11 of *p53* are shown below.

**Primer Name**	**Sequence**
Exon 2 F	TCCTGGGGGAATCCCTACAC
Exon 2 R	GGATACAGGTATGGCGGGATG
Exon 3 F	TTCCCTACTGGATGTCCCAC
Exon 3 R	CTTCTGAGACCCTGCAGTCC
Exon 4 F	TTGTTTTCCAGACTTCCTCCA
Exon 4 R	CCACTCACCGTGCACATAAC
Exon 5-6 F	ACACCTGATCGTTACTCGGC
Exon 5-6 R	GGGTTGCTAGAAAGTCAACATC
Exon 7 F	TGCCGAACAGGTGGAATATC
Exon 7 R	GGGTAGGAACCAAAGAGCGT
Exon 8 F	TCTCGGGGTTCCTGTAACTG
Exon 8 R	AGCGCTGTGGAAGGAGAGAG
Exon 9 F	AAGTCCTTTGCCCTGAACTG
Exon 9 R	CACTGAGAACCACTGTCGGA
Exon 10 F	AACCTGTAAGTGGAGCCAGC
Exon 10 R	AACCTGTAAGTGGAGCCAGC
Exon 11 F	CCTGTCTCATGGTGATGGTG
Exon 11 R	AAAAGGCAGCAGAAGGGAC

Sequencing was performed using next-generation technologies: Roche 454 GS-FLX (for *p53* exon sequencing) or Illumina HiSeq2000 (for whole-exome sequencing). Details of the calling algorithms are will be published in a separate manuscript. Briefly, substitutions were called as positions with at least 20% of reads reporting a different base with respect the reference mouse sequence (NCBIM37). Further filtering was performed by comparing the sequence to the matched normal tissue DNA as well as a pool healthy tissue DNA from large numbers of mice in the cohort.

Histochemistry, immunohistochemistry and TUNEL assay. For histopathological analysis, specimens were fixed in 4% buffered paraformaldehyde, embedded in paraffin and sectioned followed by hematoxilin and eosin staining. Acetic mucins were stained using Alcian blue solution pH 2.5 (1% Alcian blue 8GX

(Sigma-Aldrich, Deisenhofen, Germany) in 3% acetic acid). Sections were counterstained in nuclear fast red. For immunohistochemistry formalin-fixed paraffin-embedded tissue sections were dewaxed and placed in a microwave (10 min., 600 watt) to recover antigens before incubation with the following primary antibodies: p53 (NCL-p53-CM5p, Leica Micosystems, Wetzlar, Germany), p21 (C-19) (sc-397; Santa Cruz Biotechnology, Heidelberg, Germany), β-catenin (#610154, BD Biosciences, San Diego, CA), phospho-p44/42 MAPK (Erk1/2 Thr202/Tyr204) (D13.14.4E XP, Cell Signaling), Ki-67 (SP6, DCS Hamburg, Germany), c-Myc (N-262) (sc-764, Santa Cruz), cyclin D1 (SP4, Thermo Scientific, Waltham, MA) and cytokeratin 20 (GP-K20, Progen, Heidelberg, Germany). Primary antibodies were followed by secondary antibodies conjugated to biotin (Vector Laboratories, Burlingame, CA). Peroxidase conjugated streptavidin was used with 3,3'-diaminobenzidine tetrahydrochloride (DAB, Sigma-Aldrich) as chromogen for detection as described (Eser et al., 2011). For TUNEL assays, formalin fixed paraffin embedded tissue sections were dewaxed, treated with proteinase K and labeled using the In Situ Cell Death Detection POD Kit (Roche, Mannheim, Germany) as described by the manufacturer. Proliferation, apoptosis and Mapk pathway activation were quantified after Ki67, TUNEL and pERK staining, respectively. Positive cells were scored in 15-30 glands of the small and large intestine from 3-6 animals per genotype. Activation of the Wnt pathway, p53 and p21 was assessed by evaluating the presence or absence of cells positive for nuclear β-catenin, p53 and p21. For β-catenin, only nuclear β-catenin expression outside of the proliferation zone was scored as positive.

Western blotting. Tissues were homogenized in immunoprecipitation buffer containing protease and phosphatase inhibitors (Roche), normalized for protein, heated at 95°C for 5 min in Laemmli buffer and resolved on 10% SDS-polyacrylamide gels and electrophoretically transferred to polyvinylidene difluoride membranes as described. Membranes were blocked in phosphate-buffered saline supplemented with 5% skim milk and 0.1% NP-40, and incubated for 1 hour at room temperature with phospho-p44/42 MAPK (pErk1/2 Thr202/Tyr204) (D13.14.4E XP), p44/42 MAPK (Erk1/2) (L34F12), p-GSK-3ß(Ser9) (5B3), GSK-3ß (27C10) all from Cell Signaling and anti-β-actin (Sigma-Aldrich) antibodies. Proteins recognized by the antibodies were detected by the Odyssey Infrared Imaging System (Licor, Bad Homburg, Germany) using Alexa680-coupled (Molecular Probes, Leiden, Netherlands) or IRDeye800-coupled (Rockland, Gilbertsville, PA) secondary antibodies.

MSI Analysis. For examination of microsatellite instability (MSI) eight microsatellite repeat markers (Kabbarah et al., 2003; Bacher et al., 2005) were amplified by PCR using DNA isolated from hyperplastic tissue, adenomas and carcinomas as well as healthy tissue (tail tip) of the corresponding animals. 10 ng DNA was amplified with FAM labeled forward primers and reverse primers (Eurofins MWG Operon, Ebersberg, Germany) specific for the previously validated murine microsatellite markers A27, TG27, GA29, Bat67, Bat64, Bat37, Bat26, A33. Fluorescent labeled PCR products were separated and analyzed by capillary electrophoresis (ABI Prism 3100 Genetic Analyzer, Applied Biosystems, Darmstadt, Germany) and Gene Mapper software v4.1 (Applied Biosystems). Classification of microsatellite instability was performed as recommended by a National Cancer Institute workshop (Boland et al., 1998). Tumor samples with greater

or equal 40% MSI were classified as MSI-high (MSI-H), less than 40% as MSI-low (MSI-L), and samples without alterations were classified as microsatellite stable (MSS).

Drug sensitivity assays. Drug sensitivity assays were described earlier (Garnett et al., 2012).

Short term (cell viability) assay. Cancer cells were seeded into a 96 well-plate (1000 – 5000 cells per well) in 180 μl of medium. The following day, treatment was started with the specified drugs (or DMSO as negative control) at concentrations indicated in Table S13, alone or in combination with 0.5 μM PLX4720. After 6 days of treatment cell viability was determined using the Cell Titer Blue Assay (G8081, Promega), following the manufacturer's instructions. The Gefitinib concentrantions were chosen based on control experiments with EGFR-kinase domain mutant cancer cell lines from various tumor entities. These experiments showed that 0.2 μM Gefitinib completely abolished EGFR phosphorylation and downstream survival signalling (data not shown). Higher concentrations are likely to cause off-target effects.

Long term (clonogenic) assay. 2000 cells were seeded per well from a 6-well plate in 3 ml of medium. After 24 h. drugs (alone or combined) or DMSO (as negative control) were added. Drug/DMSO containing medium was changed every 3 days and cells were incubated with drugs for 10-14 days. Cells were washed in PBS and fixed by 30 min incubation in cold (4 ºC) 99% methanol with gentle rocking. Colonies were stained in 1x Modified Giemsa Stain (GS500 (20x), Sigma-Aldrich) with gentle rocking (1 h. at RT plus overnight at 4ºC). Wells were then washed in distilled water, water was removed and colonies were left to air-dry. The genetic characteristics of cell lines used and links to the COSMIC database for more detailed results are listed below:

HT29: BRAF, APC, PI3K, SMAD4, TP53, MSS
http://cancer.sanger.ac.uk/cosmic/sample/overview?id=905939
COLO-741: BRAF, CDKN2A, TP53, MSS
http://cancer.sanger.ac.uk/cosmic/sample/overview?id=906815
LS411N: BRAF, APC, FBXW7, TP53, MSI-H,
http://cancer.sanger.ac.uk/cosmic/sample/overview?id=907794
RKO: BRAF, NF1, PI3K, MSI-H
http://cancer.sanger.ac.uk/cosmic/sample/overview?id=909698
COLO-205: BRAF, APC, SMAD4, TP53, MSS
http://cancer.sanger.ac.uk/cosmic/sample/overview?id=905961
Microsatellite instability, cell line data:
http://www.sanger.ac.uk/genetics/CGP/MSI/table1.shtml

Reference List

Bacher,J.W., bdel Megid,W.M., Kent-First,M.G., and Halberg,R.B. (2005). Use of mononucleotide repeat markers for detection of microsatellite instability in mouse tumors. Mol. Carcinog. *44*, 285-292.

Boland,C.R., Thibodeau,S.N., Hamilton,S.R., Sidransky,D., Eshleman,J.R., Burt,R.W., Meltzer,S.J., Rodriguez-Bigas,M.A., Fodde,R., Ranzani,G.N., and Srivastava,S. (1998). A National Cancer Institute Workshop on Microsatellite Instability for cancer detection and familial predisposition: development of

international criteria for the determination of microsatellite instability in colorectal cancer. Cancer Res. *58*, 5248-5257.

Dankort,D., Filenova,E., Collado,M., Serrano,M., Jones,K., and McMahon,M. (2007). A new mouse model to explore the initiation, progression, and therapy of BRAFV600E-induced lung tumors. Genes Dev. *21*, 379-384.

Eser,S., Messer,M., Eser,P., von,W.A., Seidler,B., Bajbouj,M., Vogelmann,R., Meining,A., von,B.J., Algul,H., Pagel,P., Schnieke,A.E., Esposito,I., Schmid,R.M., Schneider,G., and Saur,D. (2011). In vivo diagnosis of murine pancreatic intraepithelial neoplasia and early-stage pancreatic cancer by molecular imaging. Proc. Natl. Acad. Sci. U. S. A *108*, 9945-9950.

Kabbarah,O., Mallon,M.A., Pfeifer,J.D., Edelmann,W., Kucherlapati,R., and Goodfellow,P.J. (2003). A panel of repeat markers for detection of microsatellite instability in murine tumors. Mol. Carcinog. *38*, 155-159.

Lee,E.C., Yu,D., Martinez,d., V, Tessarollo,L., Swing,D.A., Court DL, Jenkins,N.A., and Copeland,N.G. (2001). A highly efficient Escherichia coli-based chromosome engineering system adapted for recombinogenic targeting and subcloning of BAC DNA. Genomics *73*, 56-65.

Liu,P., Jenkins,N.A., and Copeland,N.G. (2003). A highly efficient recombineering-based method for generating conditional knockout mutations. Genome Res. *13*, 476-484.

Mercer,K., Giblett,S., Green,S., Lloyd,D., DaRocha,D.S., Plumb,M., Marais,R., and Pritchard,C. (2005). Expression of endogenous oncogenic V600EB-raf induces proliferation and developmental defects in mice and transformation of primary fibroblasts. Cancer Res. *65*, 11493-11500.

Pruitt,K.D., Harrow,J., Harte,R.A., Wallin,C., Diekhans,M., Maglott,D.R., Searle,S., Farrell,C.M., Loveland,J.E., Ruef,B.J., Hart,E., Suner,M.M., Landrum,M.J., Aken,B., Ayling,S., Baertsch,R., Fernandez-Banet,J., Cherry,J.L., Curwen,V., Dicuccio,M., Kellis,M., Lee,J., Lin,M.F., Schuster,M., Shkeda,A., Amid,C., Brown,G., Dukhanina,O., Frankish,A., Hart,J., Maidak,B.L., Mudge,J., Murphy,M.R., Murphy,T., Rajan,J., Rajput,B., Riddick,L.D., Snow,C., Steward,C., Webb,D., Weber,J.A., Wilming,L., Wu,W., Birney,E., Haussler,D., Hubbard,T., Ostell,J., Durbin,R., and Lipman,D. (2009). The consensus coding sequence (CCDS) project: Identifying a common protein-coding gene set for the human and mouse genomes. Genome Res. *19*, 1316-1323.
